# Fast Reconstruction of Motion‐Corrupted Data With Mobile‐GRAPPA: Motion and $\delta B_{0}$ Inhomogeneity Correction Leveraging Efficient GRAPPA

**DOI:** 10.1002/mrm.70575

**Published:** 2026-09-04

**Authors:** Yimeng Lin, Nan Wang, Daniel Abraham, Daniel Polak, Xiaozhi Cao, Aizada Nurdinova, Stephen Cauley, Kawin Setsompop

**Affiliations:** ^1^ Department of Electrical Engineering Stanford University Stanford California USA; ^2^ Department of Radiology Stanford University Stanford California USA; ^3^ Siemens Medical Solutions USA Inc. Malvern Pennsylvania USA

**Keywords:** efficient reconstruction, GRAPPA, motion correction, parallel imaging

## Abstract

**Purpose:**

To develop an accurate and computationally efficient motion‐corrected MRI reconstruction framework that incorporates hundreds to thousands of motion and $\delta B_{0}$ estimates from high‐temporal‐resolution tracking.

**Methods:**

We propose Mobile‐GRAPPA, a k‐space preprocessing approach that uses MLP‐parameterized local GRAPPA operators to jointly correct trajectory perturbations, coil reweighting, and $\delta B_{0}$‐induced phase changes before standard downstream reconstruction. Reconstruction accuracy, noise propagation, spatial resolution, and runtime were evaluated using 3D MPRAGE, multi‐echo 3D GRE, and 3D EPTI.

**Results:**

Experiments with discrete motion states demonstrated that Mobile‐GRAPPA followed by standard SENSE achieved image quality comparable to Aligned‐SENSE. In 3D GRE with 1620 tracked states and 3D EPTI with 544 tracked states, Mobile‐GRAPPA incorporated all state estimates with minimal motion‐correction overhead. Total reconstruction times were approximately 15 s for GRE and 20 min for EPTI, whereas full‐state Aligned‐SENSE was computationally prohibitive (approximately 10 h for GRE and multiple days for EPTI). Pseudo‐replica and PSF analyses showed limited additional noise amplification and negligible spatial‐resolution loss.

**Conclusion:**

Mobile‐GRAPPA enables dense motion and $\delta B_{0}$ information to be incorporated with minimal motion‐correction overhead while preserving standard SENSE, subspace, and other downstream reconstruction pipelines.

## 1 Introduction

Motion has long been a major challenge in MRI [1, 2, 3] because it simultaneously (i) perturbs k‐space sampling locations [4], (ii) alters relative coil‐sensitivity weighting [5, 6, 7, 8], and (iii) induces spatially varying polarizing magnetic field perturbations ($\delta B_{0}$) [9, 10]. These effects lead to ghosting, blurring, and coil‐weighting‐related artifacts, and are especially severe in long‐TE, $T_{2}^{*}$‐weighted acquisitions where $\delta B_{0}$ can produce large image phase perturbations.

Over the past decade, motion‐tracking technologies have advanced rapidly. External measurement systems [11, 12, 13, 14, 15, 16, 17, 18, 19, 20, 21, 22, 23, 24, 25, 26] and navigator‐based approaches [27, 28, 29, 30, 31, 32, 33, 34, 35, 36] can now provide sub‐second estimates of rigid‐body motion and, in some cases, $\delta B_{0}$ with high precision, yielding hundreds to thousands of motion states during a single scan. Here, a motion/$\delta B_{0}$ state denotes one estimation update interval over which pose and field perturbations are assumed constant, although periodic or negligible motion may produce repeated or nearly identical physical configurations. For example, in a 1‐mm whole‐brain 3D multi‐echo GRE acquisition, SMENA navigators [35] provide updates every 0.36 s, yielding 1620 motion/$\delta B_{0}$ states over a 9.7‐min scan.

However, high‐temporal‐resolution tracking introduces a new bottleneck: incorporating thousands of states into reconstruction while remaining computationally feasible. Several families of SENSE‐based reconstruction strategies have been proposed to incorporate motion information into the forward model. In Augmented‐SENSE‐type formulations [4, 37, 38], rigid motion is handled primarily through motion‐dependent k‐space encoding, typically requiring NUFFT‐based operators [39]. In contrast, Aligned‐SENSE‐type formulations [40] retain the nominal sampling trajectory and represent motion through image‐domain transformations. In these approaches, each motion state contributes a distinct encoding operator, and iterative reconstruction must repeatedly apply its forward and adjoint operators. As the number of states grows into the hundreds or thousands, the computational cost becomes prohibitive. Practical implementations therefore temporally downsample or compress motion states [38, 41], discarding expensive‐to‐acquire information. This tension becomes more acute when the motion‐free reconstruction is already computationally demanding, such as in 3D EPTI [42, 43], due to the high‐dimensional spatiotemporal encoding and accompanying subspace‐based reconstruction [44, 45]. Explicitly incorporating hundreds of motion states into the EPTI forward operator pushes computation beyond practical limits [46]. Consequently, modern acquisitions can generate more motion and $\delta B_{0}$ information than current reconstructions can afford to use.

To reduce this burden, a commonly used approximation assumes that coil sensitivity profiles move rigidly with the head and that $\delta B_{0}$ changes are negligible. Under these assumptions, a single encoding operator can be shared across all motion states, keeping the computational cost on the same order as the motion‐free reconstruction [47, 48]. While this approximation can be adequate for small motion, mild field perturbations, and short‐TE sequences, it fails under larger motion, substantial coil‐weighting variation, or pronounced susceptibility effects, particularly at higher field strengths.

These observations motivate a different strategy: keep the reconstruction operator simple and shift motion and $\delta B_{0}$ handling into a one‐time k‐space preprocessing step. Prior work has shown that local GRAPPA kernels can interpolate missing k‐space samples [49], perform gridding of non‐Cartesian data [50], and act as correction operators for eddy‐current‐induced phase errors [51, 52]. Here, we introduce Mobile‐GRAPPA, a k‐space framework that uses local GRAPPA operators to clean motion‐corrupted k‐space by correcting state‐dependent coil reweighting and $\delta B_{0}$‐induced phase, while simultaneously mapping the corrected data onto a prescribed Cartesian k‐space grid for FFT‐based downstream reconstruction. Building on recent work that jointly represents GRAPPA kernel families using a multilayer perceptron (MLP) [53], we exploit the smooth variation of optimal kernels across nearby k‐space locations and similar motion states to efficiently learn a continuous family of location‐ and state‐dependent kernels for motion‐corrupted k‐space cleaning. After this one‐time cleaning step, the data can be reconstructed using standard SENSE or more advanced pipelines, enabling fast, high‐quality reconstruction without modifying their forward operators. In extreme motion regimes, we further improve stability using a clustered variant that cleans to intermediate motion states before final reconstruction.

This work makes three contributions. First, we propose Mobile‐GRAPPA as a plug‐and‐play k‐space preprocessing module that enables fast and accurate reconstruction under motion and $\delta B_{0}$ variation. Second, we introduce an MLP‐based parameterization of the Mobile‐GRAPPA kernel family that exploits smooth structure across motion, $\delta B_{0}$, and k‐space location. Third, we propose a clustering strategy for extreme motion to improve conditioning and stability. Preliminary versions of this work were presented at ISMRM 2025 [54] and ISMRM 2026 [55].

## 2 Theory

In this section, we first review Augmented‐SENSE, approximate Augmented‐SENSE, and Aligned‐SENSE to establish the accuracy‐efficiency trade‐offs of existing motion‐aware reconstructions and to motivate Mobile‐GRAPPA. We then describe the proposed Mobile‐GRAPPA framework and its MLP‐based kernel representation.

### 2.1 Augmented‐SENSE

We denote the complex transverse magnetization in a reference pose by x(r) and the coil sensitivity maps in the same coordinate system by S(r). The acquisition is partitioned into rigid‐body motion states indexed by $n=1,\ldots ,N_{\text{state}}$, where state n is characterized by a rigid transform $T_{n}$ acting on spatial coordinates, with rotation $R_{n}$ and translation $c_{n}$. When available, each state also has an associated image phase perturbation $\varphi_{n}(r,t)$ induced by $\delta B_{0}$. We denote the measured multichannel signals by $y_{n}(t)$ and the nominal trajectory sample by $k_{n}(t)$.

Augmented‐SENSE accounts for rigid motion by transforming the sampling locations and the coil sensitivities [4, 37]. As summarized in Figure 1A, motion introduces three state‐dependent changes in the forward model: a change in sampling locations due to rotation, a change in coil weighting due to the altered pose relative to the coil array, and a change in image phase due to $\delta B_{0}$ perturbations.

**FIGURE 1:**
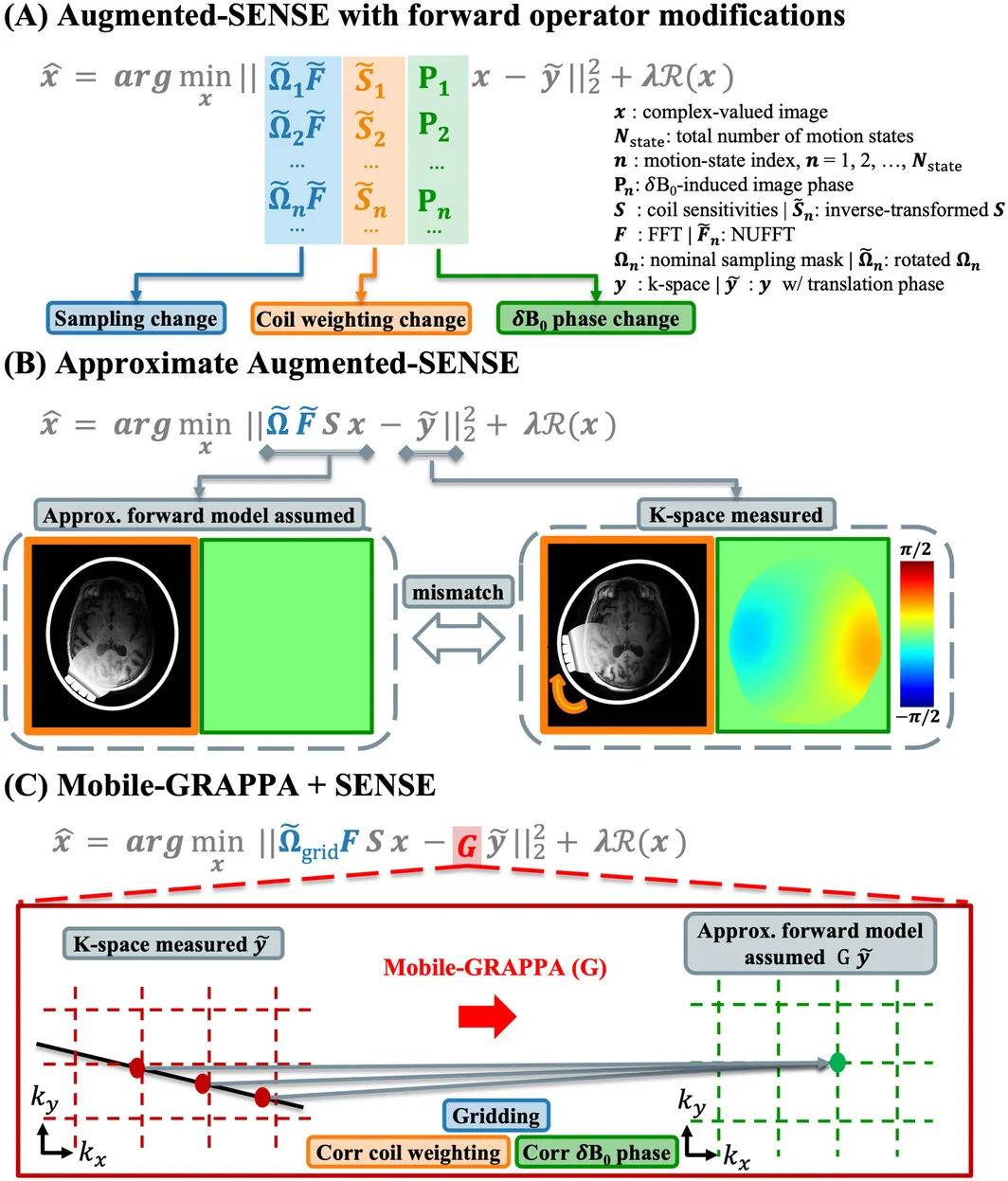
Conceptual comparison of Augmented‐SENSE, Approximate Augmented‐SENSE, and Mobile‐GRAPPA. (A) Augmented‐SENSE incorporates motion‐state‐dependent changes in the sampling coordinates, coil sensitivities, and $\delta B_{0}$‐induced image phase into the forward operator. (B) Approximate Augmented‐SENSE accounts for the motion‐dependent sampling coordinates but assumes fixed coil sensitivities and neglects the motion‐dependent $\delta B_{0}$ phase, resulting in a mismatch between the assumed forward model and the measured k‐space data. The anatomy is displayed in the reference‐image coordinates, and the apparent transformation of the coil‐sensitivity weighting represents the effective motion‐transformed sensitivity map caused by head motion relative to the fixed receive‐coil array; it does not represent physical motion of the receive coils. (C) Mobile‐GRAPPA applies the learned GRAPPA kernels to grid the measured k‐space samples while correcting motion‐dependent coil‐weighting and $\delta B_{0}$ phase changes, producing Cartesian k‐space data that are consistent with the approximate forward model used for the subsequent SENSE reconstruction. Throughout the figure, blue denotes sampling‐coordinate modification and gridding, orange denotes coil weighting changes and correction, green denotes $\delta B_{0}$ phase changes and correction, and red denotes the Mobile‐GRAPPA mapping.

First, the trajectory is inverse‐rotated:

(1) $${\tilde{k}}_{n}(t)=R_{n}^{-1}k_{n}(t),$$

which is generally not on the Cartesian grid. Therefore, Augmented‐SENSE typically requires non‐uniform Fourier transform (NUFFT) to model the Fourier encoding on ${\tilde{k}}_{n}(t)$, even when the underlying acquisition was originally Cartesian.

Second, translations are modeled as a linear phase applied to the measured signals:

(2) $${\tilde{y}}_{n}(t)=y_{n}(t)\;e^{i2\pi k_{n}(t)\cdot c_{n}}.$$

Third, coil sensitivities are inverse‐transformed into the pose of each state:

(3) $${\tilde{S}}_{n}(r)=T_{n}^{-1}\left[S(r)\right].$$

With these definitions, the Augmented‐SENSE signal model becomes

(4) $${\tilde{y}}_{n}(t)=\int \left[x(r)e^{i\varphi_{n}(r,t)}\right]{\tilde{S}}_{n}(r)\;e^{-i2\pi {\tilde{k}}_{n}(t)\cdot r}\;dr.$$

In operator form, corresponding to Figure 1A, let ${\tilde{\Omega }}_{n}$ denote the sampling operator for motion state n, $\tilde{F}$ denote the NUFFT operator, ${\tilde{S}}_{n}$ denote the coil‐encoding operator, and $P_{n}$ denote the image‐domain phase modulation induced by $\delta B_{0}$. The state‐specific encoding operator is

(5) $${\tilde{E}}_{n}={\tilde{\Omega }}_{n}\tilde{F}{\tilde{S}}_{n}P_{n}.$$

Augmented‐SENSE estimates the unknown complex‐valued image x in the reference pose from the measured k‐space data ${\tilde{y}}_{n}$ by solving

(6) $$\hat{x}=\arg\underset{x}{\min}\;\overset{N_{\text{state}}}{\underset{n=1}{\sum }}{\left\|{\tilde{E}}_{n}x-{\tilde{y}}_{n}\right\|}_{2}^{2}+\lambda ℛ(x),$$

where λ controls the regularization strength and ℛ(·) denotes the image regularizer.

Iterative solvers require repeated application of all ${\tilde{E}}_{n}$ and ${\tilde{E}}_{n}^{H}$, so the per‐iteration arithmetic cost scales approximately linearly with $N_{\text{state}}$. Moreover, storing and regenerating the state‐dependent components increases memory usage, so practical runtime can exceed the ideal linear scaling.

### 2.2 Approximate Augmented‐SENSE

To reduce computational burden, Approximate Augmented‐ SENSE (Approx‐Aug‐SENSE) retains only the trajectory inverse‐rotation and the translation phase term, while dropping explicit coil transformations and detailed $\delta B_{0}$ modeling [47, 48]. Under these approximations, ${\tilde{S}}_{n}(r)$ is replaced by a common reference sensitivity map S(r), and the phase term $e^{i\varphi_{n}(r,t)}$ is neglected:

(7) $${\tilde{y}}_{n}(t)\approx \int x(r)\;S(r)\;e^{-i2\pi {\tilde{k}}_{n}(t)\cdot r}\;dr,$$

Equivalently, all motion states share a single encoding operator,

(8) $$\tilde{E}=\tilde{\Omega }\;\tilde{F}\;S,$$

where $\tilde{\Omega }$ aggregates the inverse‐rotated sampling locations across states. Reconstruction then solves

(9) $$\hat{x}=\arg\underset{x}{\min}{\left\|\tilde{E}x-\tilde{y}\right\|}_{2}^{2}+\lambda \;ℛ(x)$$

with $\tilde{y}$ denoting the aggregated measured signals after applying the translation phase term.

This approximation removes the explicit state dependence from the forward operator and therefore avoids the near‐linear growth in per‐iteration cost with the number of motion states. However, this efficiency comes from assuming that state‐dependent coil reweighting and motion‐induced $\delta B_{0}$ phase are negligible. Figure 1B illustrates the consequence of this simplification: the assumed forward model omits the pose‐dependent coil weighting and phase variation present in the measured k‐space data, creating a mismatch between model and data. As motion, TE and field strength increase, this mismatch can become a dominant source of reconstruction error even when the sampling change itself is modeled.

### 2.3 Aligned‐SENSE

Aligned‐SENSE provides a complementary formulation that handles motion and $\delta B_{0}$ effects through image‐domain transformations rather than by modifying the k‐space trajectory [40]. Using the same notation as above, the measured signals for motion state n at readout time t are

(10) $$y_{n}(t)=\int S(r)\;T_{n}\left[x(r)e^{i\varphi_{n}(r,t)}\right]\;e^{-i2\pi k_{n}(t)\cdot r}\;dr.$$

Here, the motion operator transforms the object and $\delta B_{0}$ phase into the scanner coordinate system before multiplication by the coil sensitivities.

Aligned‐SENSE is particularly convenient for Cartesian acquisitions because it does not require trajectory modification and thus avoids replacing FFT‐based operators with NUFFT, as in the Augmented‐SENSE approach after inverse‐rotating k‐space samples. In operator form, let F denote the FFT and $\Omega_{n}$ the nominal sampling mask for state n. The state n encoding operator is

(11) $$E_{n}=\Omega_{n}\;F\;S\;T_{n}\;P_{n},$$

and reconstruction solves

(12) $$\hat{x}=\arg\underset{x}{\min}\;\overset{N_{\text{state}}}{\underset{n=1}{\sum }}{\left\|E_{n}x-y_{n}\right\|}_{2}^{2}+\lambda \;ℛ(x).$$

As in Augmented‐SENSE, iterative solvers require repeated application of every $E_{n}$ and $E_{n}^{H}$. Although the motion operator $T_{n}$ can be computed efficiently [40], the total per‐iteration cost still becomes prohibitive when hundreds to thousands of motion states are present.

### 2.4 Mobile‐GRAPPA+SENSE

This work aims to provide a generalizable reconstruction pipeline for motion‐corrupted data that maintain high accuracy with minimal additional runtime. We build on the Approx‐Aug‐SENSE forward model (Section 2.2), which achieves favorable computational scaling but, as illustrated by the mismatch in Figure 1B, no longer matches the acquired data when state‐dependent coil reweighting and $\delta B_{0}$‐induced phase are non‐negligible. We therefore introduce Mobile‐GRAPPA as a plug‐and‐play k‐space preprocessing module that removes this mismatch in a one‐time step so that the subsequent reconstruction can remain fast and unchanged.

Mobile‐GRAPPA applies a family of local GRAPPA operators {G} on the measured data with two coupled functions: (i) removal of the mismatch between Approx‐Aug‐SENSE forward operator and the acquired data by correcting state‐dependent coil reweighting and $\delta B_{0}$‐induced image phase and (ii) replacement of expensive NUFFT operations with FFTs by mapping the corrected k‐space data onto the prescribed fully sampled Cartesian $R_{1}$ grid.

The subsequent reconstruction solves

(13) $$\hat{x}=\arg\underset{x}{\min}{\left\|{\tilde{\Omega }}_{\text{grid}}\;F\;S\;x-G\;\tilde{y}\right\|}_{2}^{2}+\lambda \;ℛ(x),$$

where ${\tilde{\Omega }}_{\text{grid}}$ denotes $\tilde{\Omega }$ resampled onto the prescribed Cartesian $R_{1}$ grid.

Operationally, as shown in Figure 1C, we first apply the standard Approx‐Aug‐SENSE preprocessing for each motion state: inverse‐rotate the trajectory and apply the translation phase to the measured k‐space data. Let $k_{0}$ denote a sample location on the inverse‐rotated trajectory, and let $k_{0,\text{grid}}$ denote the nearest point to $k_{0}$ on the prescribed Cartesian $R_{1}$ grid. Mobile‐GRAPPA then uses a weighted combination of local multichannel samples around $k_{0}$ to estimate a cleaned sample at $k_{0,\text{grid}}$ with corrected coil weighting and the state‐dependent $\delta B_{0}$ phase removed. Aggregating these estimates across motion states yields Cartesian multichannel data compatible with standard SENSE or more advanced reconstructions without modifying their forward operators.

### 2.5 Mobile‐GRAPPA Training and MLP‐Based Kernel Representation

This subsection details the Mobile‐GRAPPA preprocessing module introduced in Section 2.4. Mobile‐GRAPPA is trained using low‐resolution fully sampled calibration data and then applied once to the motion‐corrupted data to generate cleaned multichannel k‐space on the prescribed $R_{1}$ Cartesian grid. As summarized in Figure 2A (a–c), the Mobile‐GRAPPA procedure has three stages: (a) preparation of calibration pairs, (b) estimation of Mobile‐GRAPPA kernel weights via an MLP, and (c) application of the trained kernels. The cleaned data are then passed to the downstream standard SENSE reconstruction shown in Figure 2A (d).

**FIGURE 2:**
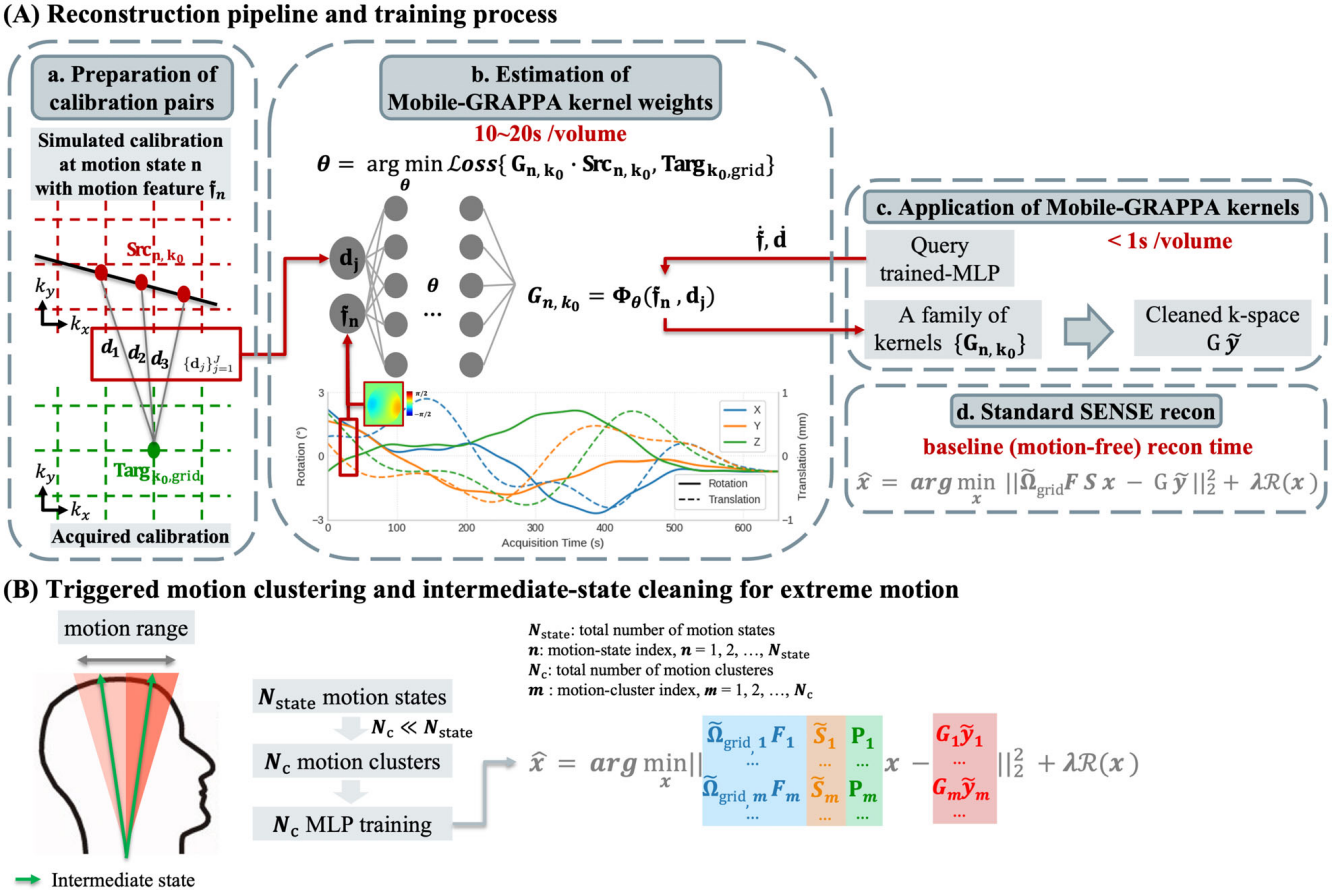
Mobile‐GRAPPA reconstruction pipeline and clustering strategy. (A) Reconstruction and training workflow. (a) Preparation of calibration pairs from low‐resolution fully sampled calibration data, where local source neighborhoods are extracted from simulated motion‐corrupted k‐space and paired with clean targets on the prescribed Cartesian grid. (b) Estimation of Mobile‐GRAPPA kernels using an MLP that maps motion‐and‐$\delta B_{0}$ features together with local source‐to‐target geometric information to a continuous family of kernels. (c) Application of the trained kernels to the acquired motion‐corrupted data to generate cleaned k‐space on the target grid. (d) Downstream reconstruction using the standard SENSE pipeline without motion‐dependent forward‐operator modification. (B) Triggered clustering for extreme motion. When motion range is large, the original motion states are grouped into a smaller number of clusters, and Mobile‐GRAPPA first cleans the data to nearby intermediate states before final reconstruction, improving conditioning and reducing noise amplification.

#### 2.5.1 Preparation of Calibration Pairs

Mobile‐GRAPPA training assumes fully sampled, low‐resolution multichannel calibration data that are free of appreciable motion and other artifacts. No motion or $\delta B_{0}$ tracking is performed during calibration acquisition. Because compatible low‐resolution calibration data are commonly acquired for coil‐sensitivity estimation or parallel‐imaging calibration, such existing data can be reused for Mobile‐GRAPPA when available, and a dedicated additional calibration scan is not necessarily required.

The calibration data are first used to estimate coil‐sensitivity maps with ESPIRiT [56] and reconstruct a coil‐combined low‐resolution reference image. The reference image is multiplied by the unmodified sensitivity maps to form the clean reference‐state multichannel target data. The target value at each grid location $k_{0,\text{grid}}$ is denoted by ${\mathrm{Targ}}_{k_{0,\text{grid}}}$.

To synthesize the source data for each motion state n, the same reference image is multiplied by sensitivity maps transformed according to the inverse rigid‐body motion, generating multichannel images with motion‐dependent coil weighting. Rigid‐body rotations are implemented using a Fourier‐interpolated shear decomposition [57], whereas translations are applied using linear phase ramps in the Fourier domain [40]. The corresponding $\delta B_{0}$‐induced phase is then applied at each echo time.

To form a source neighborhood for $k_{0}$, we select the J nearest samples along the readout dimension and denote their locations by ${\left\{k_{j}\right\}}_{j=1}^{J}$. The local source‐to‐target geometry is described by the relative offsets

(14) $$d_{j}=k_{j}-k_{0,\text{grid}},\;j=1,\ldots ,J.$$

Using the Fourier phase‐shift approach [58], each motion‐ and $\delta B_{0}$‐dependent multichannel image is multiplied by the linear phase corresponding to $d_{j}$ and then Fourier transformed. The value at $k_{0,\text{grid}}$ thereby provides the synthesized source sample at $k_{j}=k_{0,\text{grid}}+d_{j}$. The source vector ${\mathrm{Src}}_{n,k_{0}}$ is formed by stacking the synthesized multichannel samples from all J locations. In this work, J=5 source samples are included per coil. This yields the calibration pairs

(15) $$\left({\mathrm{Src}}_{n,k_{0}},{\mathrm{Targ}}_{k_{0,\text{grid}}}\right).$$

#### 2.5.2 Estimation of Mobile‐GRAPPA Kernel Weights

For each motion state n and trajectory sample $k_{0}$, we define a Mobile‐GRAPPA kernel $G_{n,k_{0}}$ that maps the local multichannel source neighborhood to the cleaned multichannel target at $k_{0,\text{grid}}$:

(16) $${\mathrm{Targ}}_{k_{0,\text{grid}}}=G_{n,k_{0}}{\mathrm{Src}}_{n,k_{0}},\;G_{n,k_{0}}\in {\mathbb{C}}^{N_{\text{coil}}\times JN_{\text{coil}}}.$$

Estimating a distinct kernel for every acquired state–location pair would require $N_{k}$ kernels per echo, where $N_{k}$ is the number of acquired k‐space samples, or $N_{k}\times N_{\text{echo}}$ kernels for multi‐echo data. Because kernel weights are correlated across nearby source‐to‐target geometries and similar poses, we instead represent the kernel family compactly using an MLP, following prior work on implicit representations of GRAPPA kernels [53].

For each calibration pair, the MLP input is formed by concatenating the local source‐to‐target geometry ${\left\{d_{j}\right\}}_{j=1}^{J}$ with the motion and/or $\delta B_{0}$ feature vector $f_{n}$. For the $\delta B_{0}$ component, we do not provide full image‐domain $\delta B_{0}$‐induced phase maps to the network. Instead, $\delta B_{0}$ is represented using spherical‐harmonic coefficients [59], which compactly describe smooth field perturbations while avoiding high‐dimensional inputs. In 3T experiments, the $\delta B_{0}$ estimates from SMENA were modeled using 2nd‐order spherical harmonics, and all 9 normalized coefficients were used directly as features. In 7T experiments, the $\delta B_{0}$ estimates required higher‐order models from st‐SMENA [60], typically 6th‐ to 8th‐order, corresponding to 49–81 coefficients. We therefore applied singular value decomposition (SVD) across the coefficient vectors and retained 6 components to preserve 99% of the energy in practice. More generally, for settings with higher‐order $\delta B_{0}$ structure, we recommend fitting up to 8th‐order spherical harmonics followed by SVD compression to 99% retained energy.

The Mobile‐GRAPPA MLP is implemented as a lightweight fully connected network with three hidden layers of width 256, ReLU activations, and a final linear output layer. The output layer produces the real and imaginary components of all complex‐valued kernel coefficients.

During training, calibration locations, trajectory locations, and motion states are stochastically sampled to form mini‐batches of source‐target pairs. Given a mini‐batch ℬ, the network is trained using the Adam optimizer with an $\ell_{1}$ data loss:

(17) $$ℒ(\theta )=\frac{1}{\left|ℬ\right|}\underset{(n,k_{0})\in ℬ}{\sum }{\left\|G_{n,k_{0}}{\mathrm{Src}}_{n,k_{0}}-{\mathrm{Targ}}_{k_{0,\text{grid}}}\right\|}_{1}.$$

The mini‐batch size was $2^{14}$ source‐target pairs, with 100 mini‐batches sampled per epoch. Training was performed for 10 epochs.

As shown in Figure 2A (b), the MLP, denoted by $\Phi_{\theta }$, learns a continuous family of kernel coefficients from the local source‐to‐target geometry and motion/$\delta B_{0}$ features, rather than fitting each kernel independently:

(18) $$G_{n,k_{0}}=\Phi_{\theta }(f_{n},\{d_{j}\}),$$

where θ denotes the trainable network parameters. In practice, MLP training requires approximately 10–20 s per volume on a single NVIDIA A5000 GPU.

#### 2.5.3 Application of Kernels

After training, we query the MLP with the motion‐and‐$\delta B_{0}$ features $f_{n}$ and local source‐to‐target offsets $\left\{d_{j}\right\}$ for each acquired trajectory sample $k_{0}$. The predicted kernel $G_{n,k_{0}}$ is then applied through (16) to produce a cleaned multichannel estimate at $k_{0,\text{grid}}$, as illustrated in Figure 2A (c).

Aggregating these outputs across all trajectory samples yields the cleaned multichannel k‐space data set $G\tilde{y}$ on the prescribed Cartesian grid. The cleaned data are then passed to the downstream reconstruction in Figure 2A (d), which uses the same standard SENSE reconstruction described in Section 2.4. In practice, this kernel‐application and cleaning step is also efficient, with typical runtimes of <1 s per volume on a single NVIDIA A5000 GPU.

### 2.6 Clustering for Extreme Motion

For moderate motion ranges, a single MLP can model kernel variation across all motion and $\delta B_{0}$ states. In extreme motion regimes or under strong pose‐dependent coil‐sensitivity variation, performance can degrade for two related reasons. First, the mapping from motion‐and‐$\delta B_{0}$ features to optimal kernel weights can vary more rapidly across a large pose range, reducing interpolation reliability for a single MLP. Second, and often more limiting, the required one‐step k‐space cleaning can become poorly conditioned: when the coil encoding is insufficiently informative for the correction implied by large motion or strong $\delta B_{0}$, the learned linear operator may amplify noise.

To improve stability and reduce noise amplification during the Mobile‐GRAPPA cleaning process, we reduce the required correction per application by cleaning the acquired data from each motion‐and‐$\delta B_{0}$ state to a nearby intermediate state rather than directly to the common reference state. For each data set, Mobile‐GRAPPA is first applied using a single MLP. If clear residual motion artifacts remain, controlled rigid‐motion simulations based on the acquired coil‐sensitivity maps are used to determine the rotational and translational limits of a single model, as characterized in Supporting Information Figure S1. The six motion parameters are normalized by these limits before k‐means clustering [61]. Starting from two clusters, the cluster count is increased until no cluster exceeds the corresponding limits, and the smallest such count is used. For each cluster, the acquired state nearest to the cluster centroid in the normalized six‐dimensional motion‐parameter space is selected as the intermediate representative state. In the only experiment requiring clustering (Experiment 1; 3T MPRAGE with a 20‐channel head‐neck coil), the empirical limits were approximately $10^{\circ }$ for each rotational component and 5 mm for each translational component.

For cluster $m\in \{1,\ldots ,N_{c}\}$, the pose of the selected representative state defines $T_{m}$, and its associated $\delta B_{0}$ estimate defines the corresponding intermediate field state. As illustrated in Figure 2B, cleaning to this intermediate state reduces the effective motion range that each Mobile‐GRAPPA kernel set must correct. A cluster‐specific MLP is trained using motion relative to $T_{m}$ and the corresponding $\delta B_{0}$ features.

The intermediate‐state cleaning operation produces a cleaned estimate on the $R_{1}$ grid consistent with the intermediate pose:

(19) $${\mathrm{Targ}}_{m,k_{0,\text{grid}}}=G_{m,k_{0}}\;{\mathrm{Src}}_{n,k_{0}},$$

where ${\mathrm{Targ}}_{m,k_{0,\text{grid}}}$ denotes the desired multichannel sample at $k_{0,\text{grid}}$ with coil weighting and $\delta B_{0}$‐induced phase referenced to intermediate pose m.

After cleaning, we aggregate samples within each intermediate state to form $N_{c}$ gridded data sets and perform reconstruction using a small set of discrete states rather than the full set of original motion states. The encoding operator for intermediate state m is:

(20) $${\tilde{E}}_{m}={\tilde{\Omega }}_{\text{grid},m}\;F\;{\tilde{S}}_{m}\;P_{m},$$

and the reconstruction solves:

(21) $$\hat{x}=\arg\underset{x}{\min}\;\overset{N_{c}}{\underset{m=1}{\sum }}{\left\|{\tilde{E}}_{m}x-G_{m}\;{\tilde{y}}_{m}\right\|}_{2}^{2}+\lambda \;ℛ(x).$$

This strategy reduces $N_{\text{state}}$ tracked states to $N_{c}\ll N_{\text{state}}$ intermediate states, so downstream reconstruction scales primarily with $N_{c}$. The trade‐off is that $N_{c}$ cluster‐specific MLPs must be trained and $N_{c}$ intermediate states reconstructed. Thus, clustered Mobile‐GRAPPA is more expensive than the standard single‐model pipeline but remains substantially cheaper than full‐state Aligned‐SENSE or Augmented‐SENSE.

## 3 Methods

We performed six primary in vivo experiments to evaluate Mobile‐GRAPPA for removing motion‐induced k‐space inconsistencies while preserving the simplicity of standard downstream reconstructions. Across experiments, Mobile‐GRAPPA used the same classes of inputs: fully sampled low‐resolution calibration data, motion and $\delta B_{0}$ estimates, the k‐space sampling trajectory, and motion‐corrupted k‐space data. In experiments involving dynamic tracking, motion and $\delta B_{0}$ estimates were obtained using SMENA [35] or st‐SMENA [60], with experiment‐specific temporal resolution described below. All in vivo studies were approved by the institutional review board, and written informed consent was obtained from all subjects prior to scanning. The data for Experiments 1–3 were acquired from three different healthy volunteers, whereas those for Experiments 4–6 were acquired from a fourth healthy volunteer.

Experiments 1–3 evaluate Mobile‐GRAPPA using Aligned‐SENSE as an algorithmic reference with shared limited numbers of discrete motion and/or $\delta B_{0}$ states. The corresponding NRMSE therefore measures agreement between reconstruction strategies rather than absolute error. Experiments 4–6 evaluate computational feasibility with dense tracking, for which full‐state Aligned‐SENSE is prohibitively expensive, so state‐compressed Aligned‐SENSE was used as a practical benchmark. Independent stationary‐reference validation was additionally performed using separate motion and stationary acquisitions with the Experiment 4 3D GRE and Experiment 5 3D EPTI protocols, as shown in Supporting Information Figures S2 and S3, respectively. Table 1 summarizes the six primary experiments.

**TABLE 1:** In vivo experiment protocols.

Exp.	Sequence	Field	FOV (mm 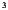 )	Resolution (mm 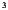 )	TA (min)	TE/TI/TR (ms)	# of Echoes	# of States
1	MPRAGE	3T	256×256×192	1.26×1.0×1.0	2.5/pose	2.19/1000/2300	1	12
2	multi‐echo GRE	3T	240×240×216	1.0×1.0×1.0	9.7	10.3‐43.1/‐/45	14	12
3	multi‐echo GRE	7T	240×240×216	1.0×1.0×1.0	9.7	6.2‐30.3/‐/45	8	12
4	multi‐echo GRE	3T	240×240×216	1.0×1.0×1.0	9.7	6‐29.2/‐/45	8	1620
5	EPTI	3T	240×240×216	1.0×1.0×1.0	2.2	10.2‐60.7/‐/60	48	544
6	EPTI	3T	240×240×216	1.0×1.0×1.0	2.2	10.2‐60.7/‐/60	48	544

### 3.1 Calibration Data Acquisition

For Experiment 1, Mobile‐GRAPPA used the conventional ACS reference data acquired within the MPRAGE measurement, forming a 30×30 calibration block along $k_{y}$ and $k_{z}$. For Experiments 2–6, an additional fully sampled, low‐resolution 3D GRE calibration scan was acquired with 46 $k_{y}$ lines and 42 $k_{z}$ partitions. In all experiments, the calibration data were acquired with the same receiver coil configuration and FOV prescription as the corresponding main acquisition, were fully sampled along the readout direction, and had an acquisition time of approximately 6 s.

### 3.2 Experiment 1: Coil Reweighting Correction Under Discretized Static Poses (3D MPRAGE, 3T)

Experiment 1 used 3D MPRAGE to isolate the effect of motion‐induced coil weighting change, while minimizing $\delta B_{0}$ phase accrual because of the short TE. Data were acquired on a 3T MR scanner (MAGNETOM Vida; Siemens Healthineers, Forchheim, Germany) using a 20‐channel head‐neck coil. Acceleration was R=4 using a 2×2 CAIPI undersampling pattern [62]. Five separate scans were performed at different static head poses and retrospectively combined by mixing k‐space segments across poses to form a synthesized motion‐corrupted data set with 12 motion states, with three of the five acquired poses each contributing to three motion states, one contributing to two motion states, and one contributing to one motion state. Rigid motion parameters were estimated by registering each pose image to a reference pose. Mobile‐GRAPPA cleaned the mixed‐pose k‐space to the reference pose, after which the cleaned data were reconstructed using standard SENSE (Mobile‐GRAPPA + SENSE). Reconstructions were compared against no correction, Approx‐Aug‐SENSE and Aligned‐SENSE.

### 3.3 Experiment 2: $\delta B_{0}$‐Induced Phase Correction Under Step Motion (Multi‐Echo 3D GRE, 3T)

Experiment 2 used multi‐echo 3D GRE to evaluate Mobile‐GRAPPA's ability to correct both motion and $\delta B_{0}$‐induced phase and to assess its robustness across echo times. Data were acquired on a 3T MR scanner (GE UHP; GE Healthcare, Milwaukee, WI, USA) using a 32‐channel head coil. Acceleration was R=4 using a 2×2 uniform undersampling pattern. A healthy volunteer performed stepwise head motion during the acquisition, yielding 12 discrete motion states. Mobile‐GRAPPA cleaning used motion and $\delta B_{0}$ estimates from SMENA navigators every 50 s. Because the $\delta B_{0}$‐induced phase evolves with echo time, a separate set of Mobile‐GRAPPA kernels was trained and applied for each echo. Mobile‐GRAPPA was evaluated in three configurations: coil‐weighting‐only correction, $\delta B_{0}$‐only correction, and joint correction. The cleaned data were reconstructed using standard SENSE. Reconstructions were compared against no correction, Approx‐Aug‐SENSE, and Aligned‐SENSE.

### 3.4 Experiment 3: High Field Robustness Under Step Motion (Multi‐Echo 3D GRE, 7T)

Experiment 3 repeated the multi‐echo 3D GRE characterization at 7T, where susceptibility‐related $\delta B_{0}$ perturbations and motion‐induced changes in effective coil weighting are expected to be more pronounced than at 3T [9, 59, 63]. Data were acquired on a 7T MR scanner (MAGNETOM Terra.X; Siemens Healthineers, Forchheim, Germany) using a 32‐channel head coil. A healthy volunteer moved between six stationary head poses during one scan. Motion and $\delta B_{0}$ were tracked densely by st‐SMENA navigators every 0.36 s. The tracked motion and $\delta B_{0}$ estimates were compressed into 12 representative states spanning the six stationary poses and the intervening transition periods, which kept the Aligned‐SENSE reference reconstruction tractable.

### 3.5 Experiment 4: Feasibility Under Continuous Free Motion and Dense Tracking (Multi‐Echo 3D GRE, 3T)

Experiment 4 evaluates Mobile‐GRAPPA's scalability with high‐temporal‐resolution tracking using the same scanner and spatial resolution as Experiment 2, with an eight‐echo multi‐echo 3D GRE acquisition. The subject moved freely throughout the 9.7‐min scan while SMENA provided motion and $\delta B_{0}$ estimates every 0.36 s, yielding $N_{\text{state}}=1620$ motion‐and‐$\delta B_{0}$ states. To broaden the evaluation across volunteers and motion patterns, the acquisition was repeated in three additional healthy volunteers with small, moderate, and large continuous motion, as shown in Supporting Information Figure S4. Mobile‐GRAPPA cleaned the k‐space using all tracked states and the cleaned data were reconstructed using standard SENSE. Aligned‐SENSE was evaluated with temporal down‐sampling or motion‐state compression because the approximately 10 h runtime at $N_{\text{state}}=1620$ made direct evaluation impractical for routine comparison.

### 3.6 Experiment 5: Feasibility When Motion‐Free Reconstruction is Computationally Expensive (3D EPTI, 3T)

Experiment 5 tested Mobile‐GRAPPA on the same scanner as Experiment 2 but with 1 mm isotropic 3D Echo Planar Time‐resolved Imaging (EPTI) [42, 43]. EPTI performs efficient spatiotemporal k‐t sampling with subspace reconstruction to recover distortion‐ and blurring‐free time‐resolved $T_{2}^{*}$‐weighted images, making even the motion‐free reconstruction computationally demanding [45]. The subject moved freely during the 2.2‐min acquisition while SMENA provided motion and $\delta B_{0}$ estimates every 0.24 s, yielding $N_{\text{state}}=544$ tracked states. Mobile‐GRAPPA cleaned the k‐space using all tracked states and the cleaned data were reconstructed using the standard EPTI subspace reconstruction pipeline. Explicit motion modeling with an Aligned‐SENSE‐style EPTI forward operator was evaluated using down‐sampling or motion‐state compression due to prohibitive runtime scaling.

### 3.7 Experiment 6: Feasibility Under Deep Breathing and Dense Tracking (3D EPTI, 3T)

Experiment 6 isolates respiration‐driven $\delta B_{0}$ perturbations by instructing the subject to minimize head motion and perform deep breathing. Acquisition and reconstruction settings were matched to Experiment 5 to attribute differences primarily to time‐varying $\delta B_{0}$.

### 3.8 Quantitative Evaluation

For Experiments 1–3, residual artifacts were assessed using 5×‐scaled error maps and normalized root‐mean‐square error (NRMSE) relative to the corresponding Aligned‐SENSE reconstruction.

Noise amplification introduced by Mobile‐GRAPPA was characterized using 200 pseudo‐replicas [64] in Experiments 2 and 3. After prewhitening with the coil‐noise covariance matrix, i.i.d. complex Gaussian noise was added to the k‐space inputs for Mobile‐GRAPPA + SENSE and Aligned‐SENSE. The resulting empirical 1/g‐factor maps quantify additional noise amplification from Mobile‐GRAPPA cleaning relative to Aligned‐SENSE, rather than the parallel‐imaging g‐factor of the downstream SENSE reconstruction.

Spatial resolution was evaluated using a numerical point‐spread‐function (PSF) simulation based on the Experiment 4 motion trajectory and corresponding $\delta B_{0}$ maps compressed into 12 representative states. Unit delta functions at four spatial locations were reconstructed using Aligned‐SENSE and Mobile‐GRAPPA. The FWHM was measured along x, y, and z through each reconstructed PSF peak using linear interpolation at the half‐maximum crossings.

## 4 Results

### 4.1 Discretized‐State Characterization (Experiments 1–3)

In the discretized MPRAGE experiment, Mobile‐GRAPPA effectively corrected large inconsistencies arising from substantial motion, with the rotational range exceeding $10^{\circ }$ (Figure 3A). The no‐correction reconstruction exhibits severe motion artifacts, while Approx‐Aug‐SENSE reduces these artifacts but still leaves errors consistent with pose‐dependent coil reweighting. Mobile‐GRAPPA substantially suppresses these inconsistencies and approaches the Aligned‐SENSE reference in both axial and sagittal views, with little remaining structure in the 5× error maps. Quantitatively, NRMSE decreases from 56% to 63% (no correction) to 4%–5% with Mobile‐GRAPPA and further to 3% with two‐cluster Mobile‐GRAPPA. The single‐model Mobile‐GRAPPA reconstruction incurs only a small fixed training overhead relative to baseline reconstruction, whereas the two‐cluster version doubles the training time and increases the reconstruction time from 5 s to 15 s.

**FIGURE 3:**
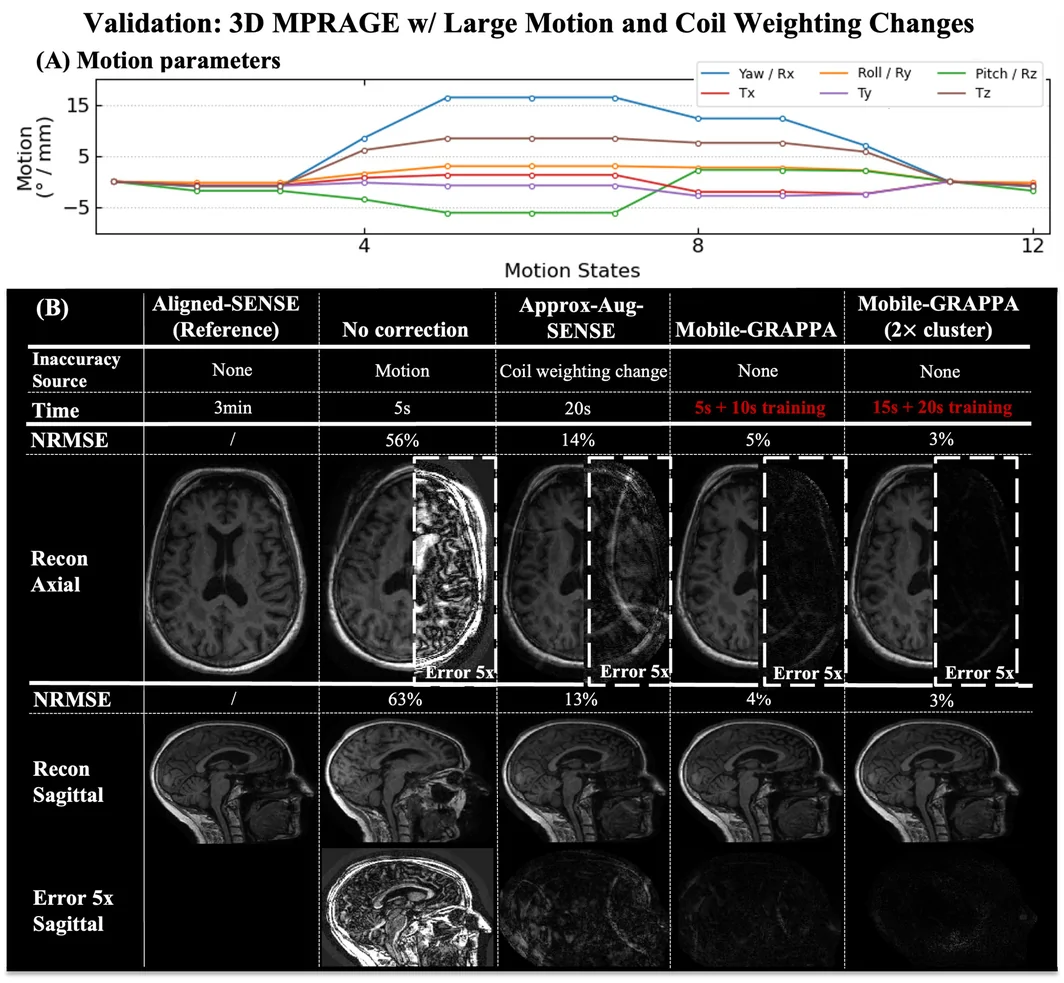
Experiment 1: synthesized 3D MPRAGE at 3T with large discrete motion and coil weighting changes. (A) Six rigid‐body motion parameters across the 12 motion states. (B) Reconstruction comparison. Column 1: Aligned‐SENSE reference. Column 2: no correction. Column 3: Approx‐Aug‐SENSE. Column 4: Mobile‐GRAPPA + SENSE. Column 5: two‐cluster Mobile‐GRAPPA + SENSE. Axial and sagittal reconstructions are shown together with 5× error maps relative to Aligned‐SENSE. Mobile‐GRAPPA substantially reduces motion‐related inconsistencies and approaches the Aligned‐SENSE reference while maintaining near‐baseline reconstruction time.

The step‐motion multi‐echo GRE experiment at 3T was used to separate the residual effects of pose‐dependent coil‐weighting changes and $\delta B_{0}$ phase errors. Without correction, the NRMSE was 12% at the early echo (TE = 10 ms) and 15% at the later echo (TE = 25 ms). Approx‐Aug‐SENSE reduced the NRMSE to 3% and 6%, respectively, but residual artifacts remained, particularly at the later echo (Figure 4B). Mobile‐GRAPPA with coil‐weighting correction only, while leaving the $\delta B_{0}$ phase uncorrected, resulted in NRMSE values of 3% at TE = 10 ms and 5% at TE = 25 ms, representing only a modest improvement over Approx‐Aug‐SENSE in this 3T experiment. In contrast, Mobile‐GRAPPA with $\delta B_{0}$ phase correction only, while leaving pose‐dependent coil weighting uncorrected, reduced the NRMSE to 2% at both echo times and more effectively suppressed the residual ringing‐like artifacts. Joint correction of both effects also resulted in an NRMSE of 2% at both echo times and produced the complete Mobile‐GRAPPA reconstruction. These results indicate that $\delta B_{0}$ phase errors were the dominant residual error source in this experiment, with their effect becoming more pronounced at the later echo, whereas pose‐dependent coil‐weighting changes made a smaller secondary contribution.

**FIGURE 4:**
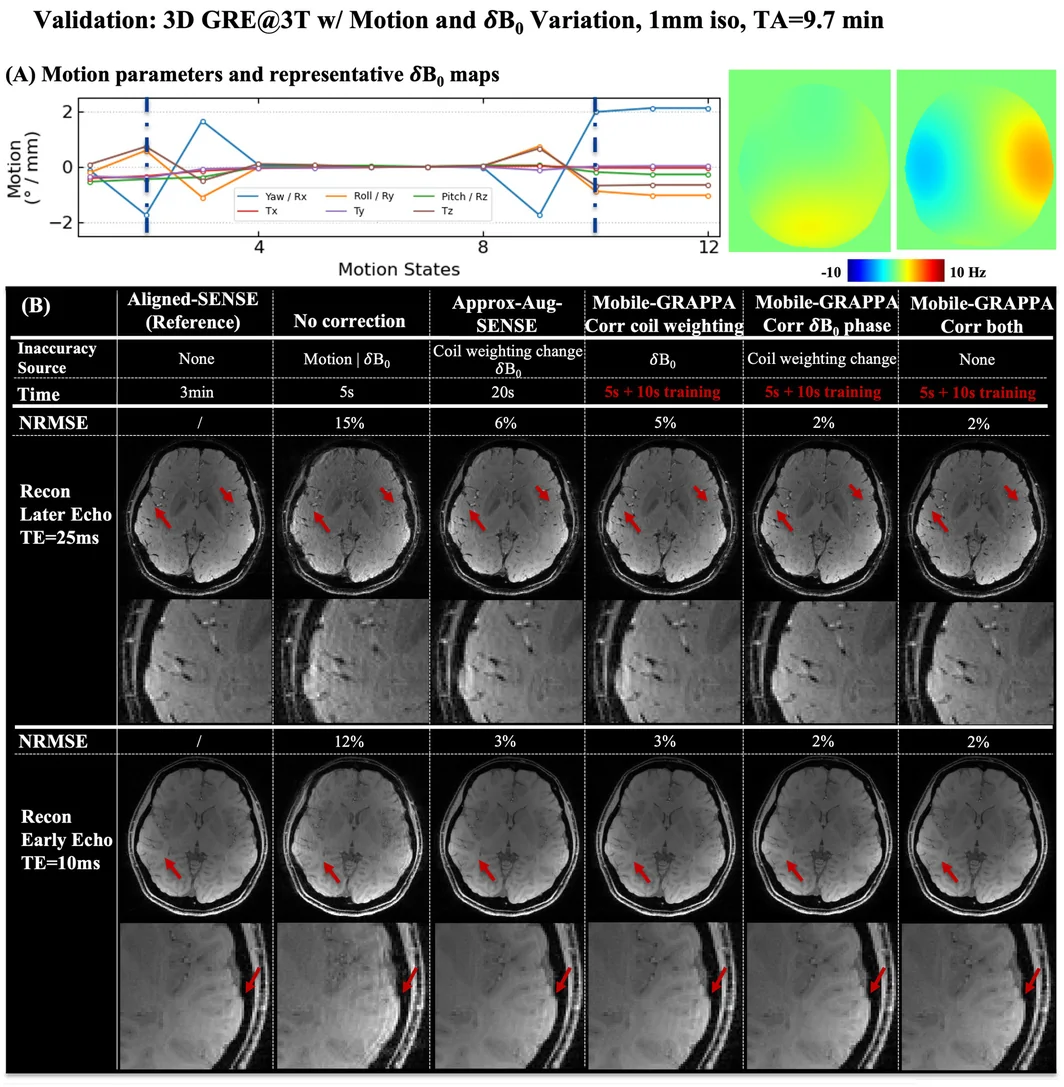
Experiment 2: step‐motion multi‐echo 3D GRE at 3T with motion and $\delta B_{0}$ variation. (A) Six rigid‐body motion parameters across the 12 motion states, with representative $\delta B_{0}$ maps shown for selected states. (B) Reconstruction comparison at an early echo (TE = 10 ms) and a later echo (TE = 25 ms). Column 1: Aligned‐SENSE reference. Column 2: no correction. Column 3: Approx‐Aug‐SENSE. Column 4: Mobile‐GRAPPA with coil‐weighting correction only, leaving $\delta B_{0}$ phase uncorrected. Column 5: Mobile‐GRAPPA with $\delta B_{0}$ phase correction only, leaving pose‐dependent coil weighting uncorrected. Column 6: Mobile‐GRAPPA jointly correcting both coil weighting and $\delta B_{0}$ phase. Red arrows indicate representative residual artifacts.

Figure 5 demonstrates that ultra‐high‐field motion correction requires joint handling of coil reweighting and $\delta B_{0}$ phase. At 7T, Approx‐Aug‐SENSE correction leaves pronounced residual artifacts, consistent with stronger pose‐dependent coil weighting variation and increased susceptibility‐driven field perturbations (Figure 5B). Coil‐only and $\delta B_{0}$‐only Mobile‐GRAPPA remove their targeted error sources but leave complementary residual artifacts, whereas joint correction produces the cleanest reconstructions in both axial and sagittal views. Because the Aligned‐SENSE reference can contain residual artifacts, the NRMSE values in Figure 5 only quantify agreement with this algorithmic reference rather than absolute image accuracy. These results indicate that both correction components are required to reduce motion‐induced artifacts at ultra‐high field.

**FIGURE 5:**
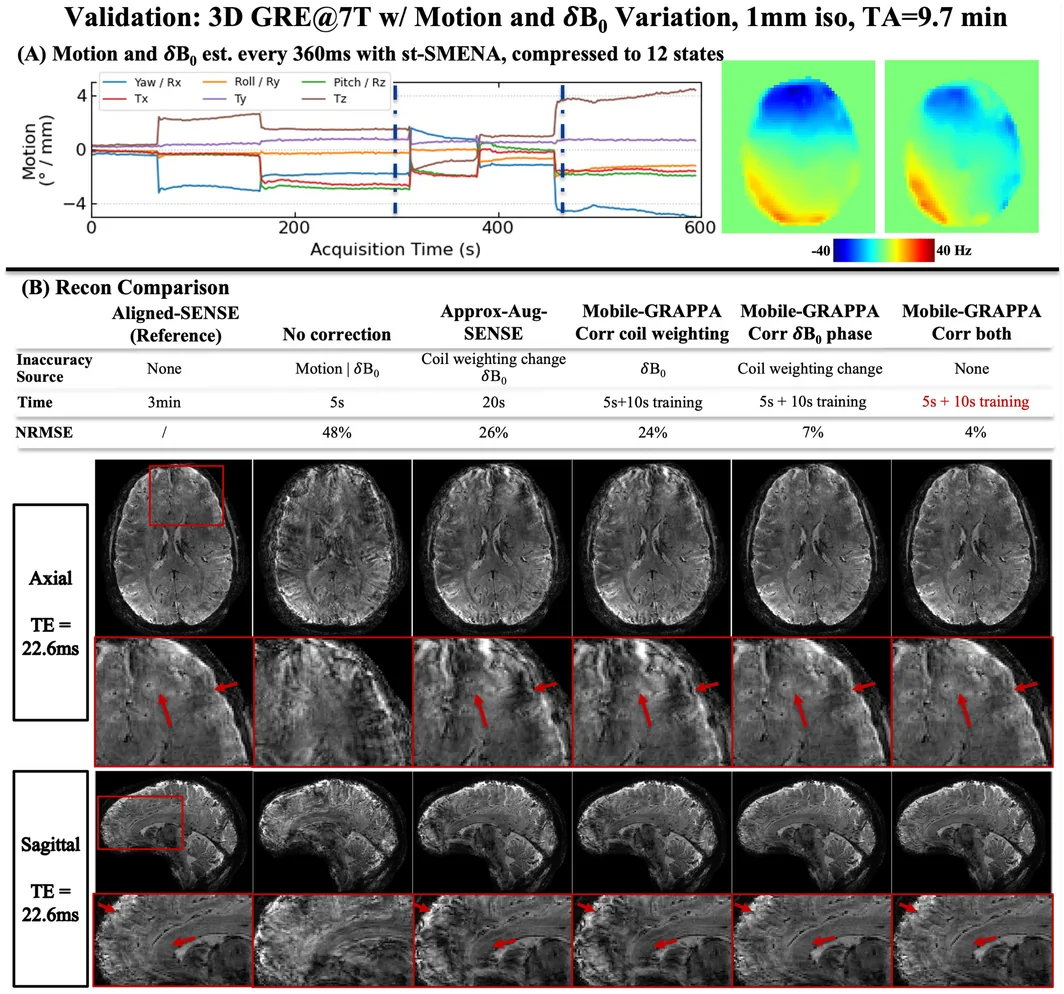
Experiment 3: step‐motion multi‐echo 3D GRE at 7T with stronger motion‐ and susceptibility‐related effects. (A) Motion and $\delta B_{0}$ estimates tracked by st‐SMENA every 360 ms and compressed to 12 representative states for tractable Aligned‐SENSE reconstruction. (B) Reconstruction comparison at TE = 22.6 ms. Column 1: Aligned‐SENSE reference. Column 2: no correction. Column 3: Approx‐Aug‐SENSE. Column 4: Mobile‐GRAPPA correcting coil weighting only. Column 5: Mobile‐GRAPPA correcting $\delta B_{0}$ phase only. Column 6: Mobile‐GRAPPA jointly correcting both coil weighting and $\delta B_{0}$. Zoomed axial and sagittal views highlight residual artifacts. At 7T, joint correction is required to approach the Aligned‐SENSE reference.

### 4.2 Quantitative Characterization

Figure 6 quantitatively compares reconstruction error, noise behavior, and spatial resolution. In Figure 6A, NRMSE increased with TE at both 3T and 7T, particularly without $\delta B_{0}$ correction. Mobile‐GRAPPA with $\delta B_{0}$ correction substantially reduced error across echoes. Explicit coil‐weighting correction provided little additional benefit at 3T but a clearer improvement at 7T. These trends are consistent with greater off‐resonance phase accumulation at longer TE and higher field strength, and with stronger motion‐induced changes in effective coil weighting from more spatially varying coil sensitivities [63]. Representative coil sensitivity magnitude maps and $\delta B_{0}$ maps at both field strengths are shown in Supporting Information Figure S5.

**FIGURE 6:**
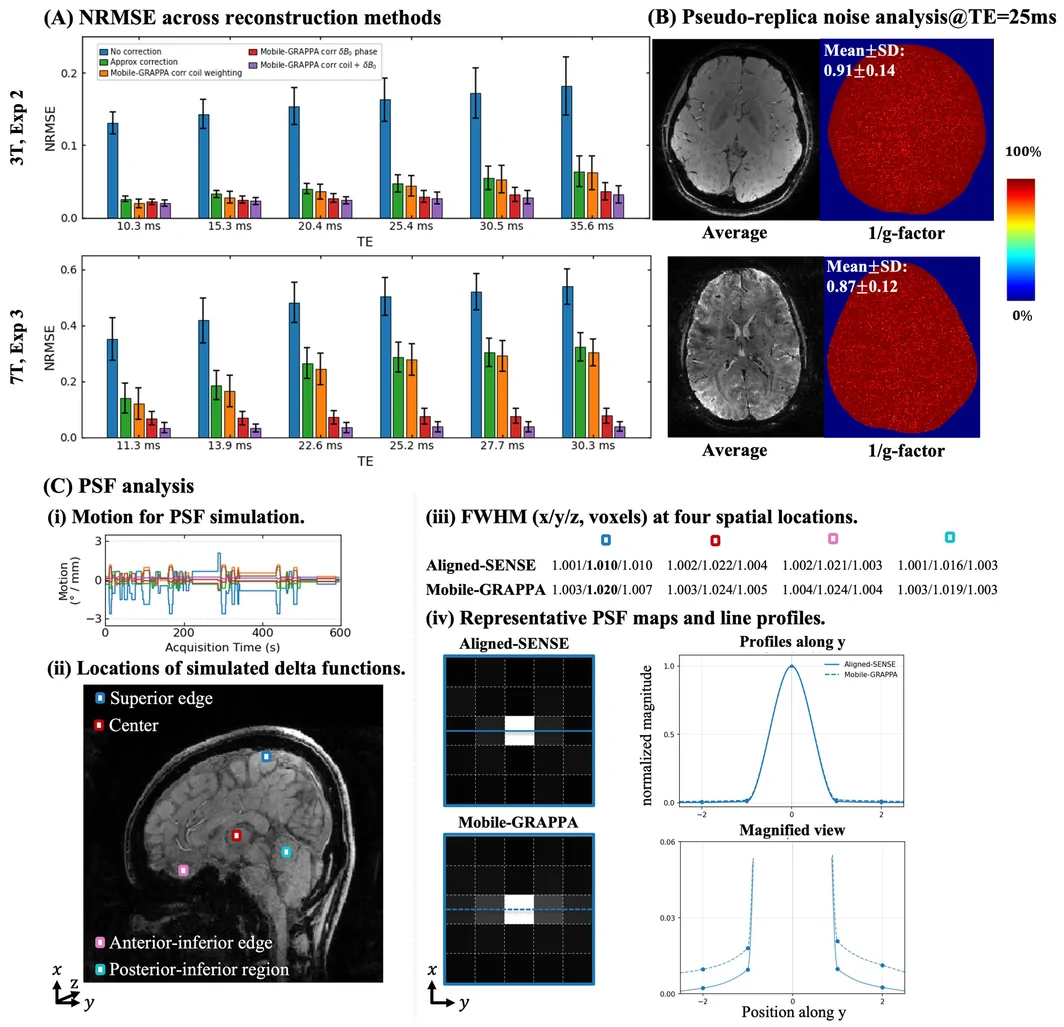
Quantitative evaluation of reconstruction accuracy, noise propagation, and spatial resolution. **(A)** Mean slice‐wise NRMSE across axial slices versus echo time at 3T in Experiment 2 and at 7T in Experiment 3. Error bars indicate the standard deviation across slices. **(B)** Pseudo‐replica analysis at TE=25ms, showing average reconstructed images and empirical 1/g‐factor maps relative to Aligned‐SENSE. Values indicate the mean and standard deviation within the brain mask. **(C)** Numerical PSF analysis using the motion trajectory and corresponding $\delta B_{0}$ maps from Experiment 4, compressed into 12 representative states: **(i)** motion trajectory; **(ii)** locations of the four simulated unit delta functions; **(iii)** FWHM along the x, y, and z directions; and **(iv)** representative normalized PSF maps and y‐direction profiles at the superior‐edge location. Bold values in **(iii)** identify the y‐direction FWHM measurements shown in **(iv)**. Markers denote the original voxel samples, and curves in the magnified view are interpolated for visualization.

At TE=25ms, pseudo‐replica analysis yielded spatial mean ± standard deviation values of the 1/g‐factor of 0.91±0.14 at 3T and 0.87±0.12 at 7T relative to Aligned‐SENSE (Figure 6B), indicating small but measurable additional noise amplification from Mobile‐GRAPPA cleaning.

In the PSF analysis (Figure 6C), FWHM values were 1.001–1.022 voxels for Aligned‐SENSE and 1.003–1.024 voxels for Mobile‐GRAPPA, with a maximum matched‐pair increase of 0.010 voxel. The similar main‐lobe widths indicate negligible spatial‐resolution loss with Mobile‐GRAPPA.

### 4.3 Dense Tracking: Feasibility and Runtime Scaling (Experiments 4–6)

The free‐motion 3T GRE experiment demonstrates the scaling limitation of explicit state‐dependent reconstruction. With 1620 tracked motion‐and‐$\delta B_{0}$ states, Aligned‐SENSE required temporal down‐sampling or state compression, producing a visible quality–runtime trade‐off. Mobile‐GRAPPA instead used all 1620 states in a one‐time cleaning step followed by standard SENSE (Figure 7). It suppressed early‐echo motion ringing and later‐echo $\delta B_{0}$‐related artifacts while maintaining near‐baseline reconstruction time with only a small training overhead.

**FIGURE 7:**
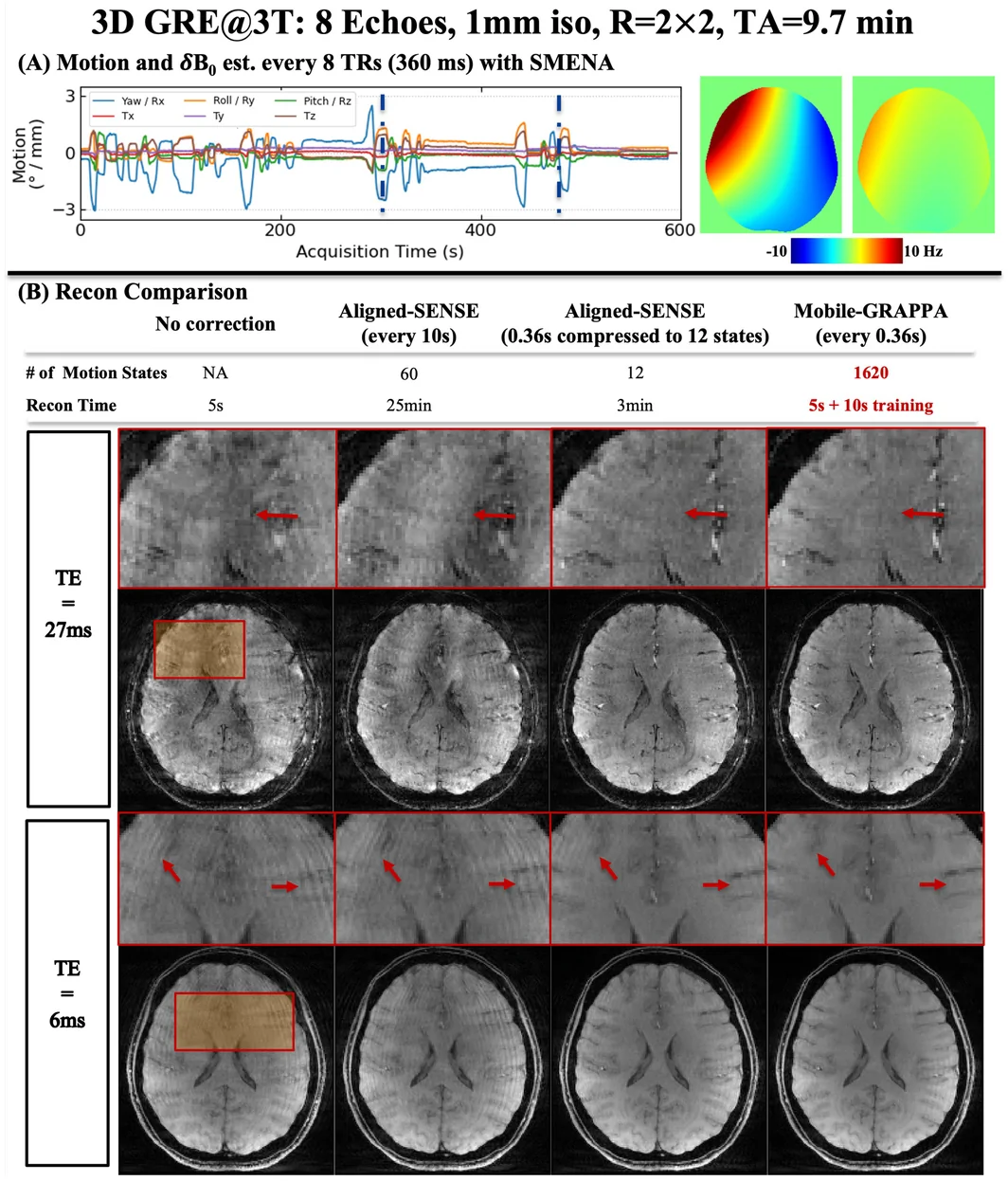
Experiment 4: dense motion and $\delta B_{0}$ tracking in 1‐mm isotropic multi‐echo 3D GRE at 3T. (A) Motion and $\delta B_{0}$ estimates obtained every 360 ms using SMENA. (B) Reconstruction comparison. Column 1: no correction. Column 2: Aligned‐SENSE using temporally downsampled motion updates (approximately every 10 s). Column 3: Aligned‐SENSE using 12 representative states obtained by k‐means compression from the dense motion estimates. Column 4: Mobile‐GRAPPA using all 1620 tracked motion‐and‐$\delta B_{0}$ states. Comparisons are shown for an early echo (TE = 6 ms) and a later echo (TE = 27 ms). Mobile‐GRAPPA preserves dense motion information while maintaining near‐baseline reconstruction time plus a small one‐time training overhead.

The computational advantage is greater for EPTI, whose motion‐free reconstruction is already expensive. With 544 tracked states, explicit state‐dependent modeling remained prohibitively slow even after state compression, whereas Mobile‐GRAPPA used all states and preserved the standard subspace reconstruction (Figure 8). The resulting PD and $T_{2}^{*}$ outputs showed reduced artifacts, while the total runtime remained dominated by the baseline EPTI reconstruction rather than motion correction.

**FIGURE 8:**
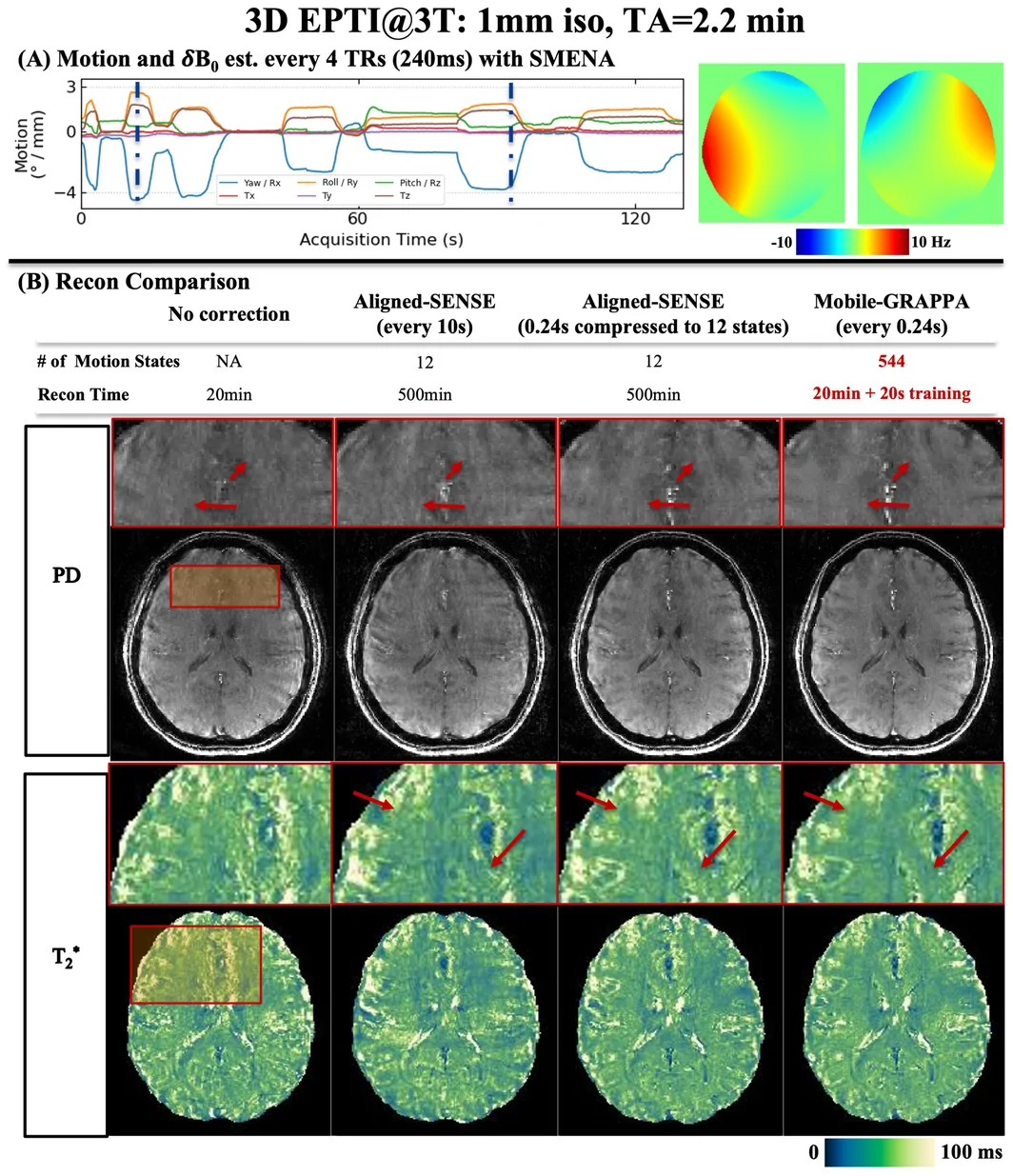
Experiment 5: 3D‐EPTI at 3T with free motion and dense tracking. (A) Motion and representative $\delta B_{0}$ maps estimated every 240 ms using SMENA. (B) Reconstruction comparison for proton‐density (PD) and $T_{2}^{*}$ outputs. Column 1: no correction. Column 2: Aligned‐SENSE using temporally downsampled motion updates (approximately every 10 s). Column 3: Aligned‐SENSE using 12 representative states obtained by k‐means compression from the dense motion estimates. Column 4: Mobile‐GRAPPA using all 544 tracked motion‐and‐$\delta B_{0}$ states. Red arrows indicate representative residual artifacts. Mobile‐GRAPPA improves image and parametric‐map quality while preserving the baseline EPTI reconstruction pipeline and avoiding the prohibitive runtime of explicitly state‐dependent reconstruction.

Under deep breathing with minimal intended rigid motion, Mobile‐GRAPPA also reduced respiration‐related artifacts in the multi‐echo images and improved the derived PD, $T_{2}^{*}$, and QSM contrasts, particularly in susceptibility‐sensitive regions (Figure 9). This result demonstrates correction of time‐varying $\delta B_{0}$ dynamics without modifying the downstream EPTI reconstruction.

**FIGURE 9:**
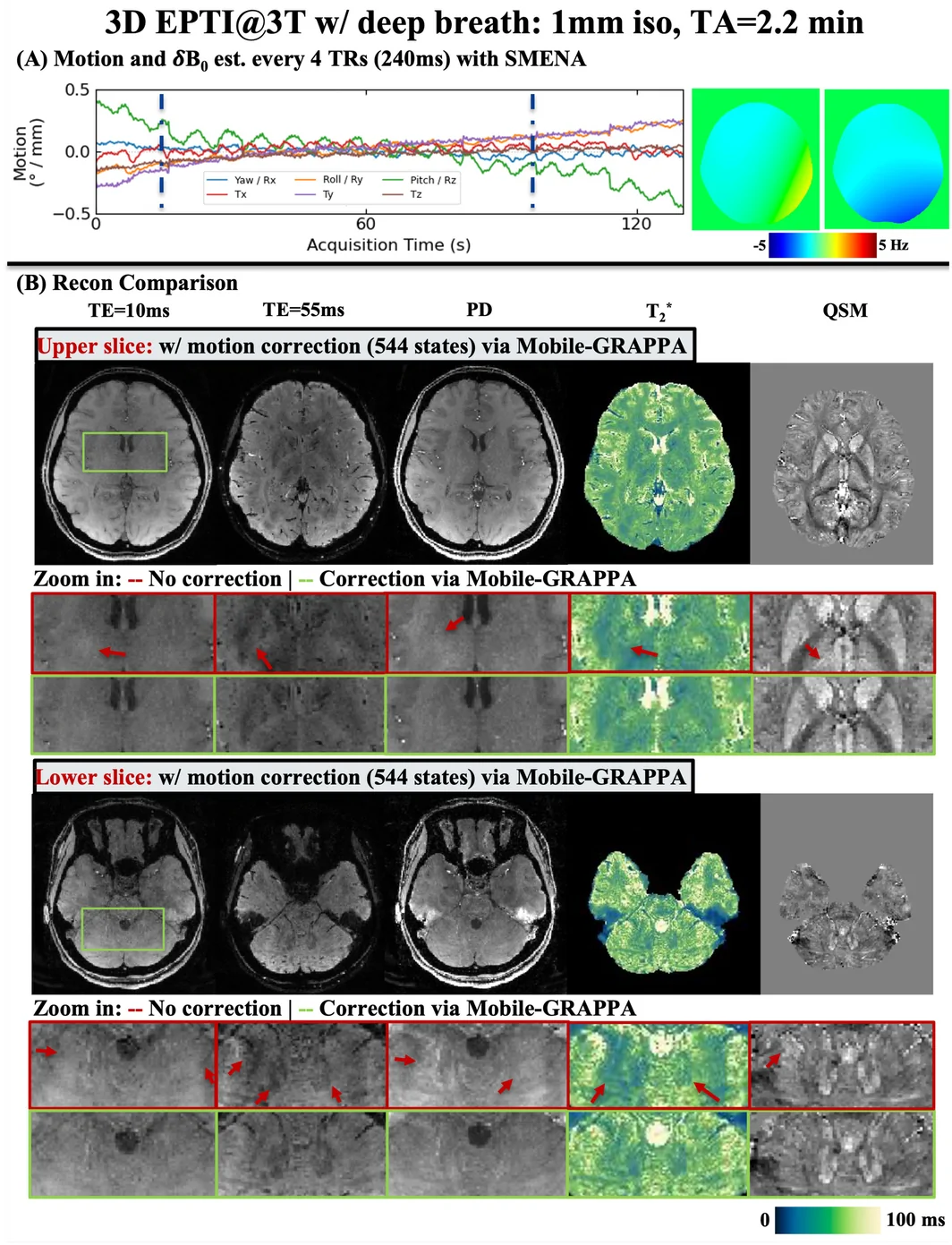
Experiment 6: 3D‐EPTI at 3T under deep breathing with minimal intended rigid motion. (A) Motion traces and representative $\delta B_{0}$ maps estimated every 240 ms using SMENA. (B) Comparison of reconstructed multi‐echo images, PD, $T_{2}^{*}$, and QSM [65] with and without Mobile‐GRAPPA correction using all 544 tracked states. Red boxes indicate zoomed regions and arrows highlight representative breathing‐related artifacts that are reduced after Mobile‐GRAPPA cleaning. The results show that Mobile‐GRAPPA remains effective in a $\delta B_{0}$‐dominant regime and improves both image quality and derived quantitative contrasts without modifying the downstream EPTI reconstruction.

## 5 Discussion

The main advantage of Mobile‐GRAPPA is that it moves dense motion and $\delta B_{0}$ modeling out of the iterative image‐space reconstruction. Learned local GRAPPA operators map motion‐ and $\delta B_{0}$‐corrupted k‐space to a common reference state, after which reconstruction proceeds using standard SENSE or another downstream pipeline. Motion and $\delta B_{0}$ modeling are therefore decoupled from the image‐space solve.

Across the discrete‐state experiments, Mobile‐GRAPPA achieved image quality comparable to Aligned‐SENSE, including at 7T, where $\delta B_{0}$ and coil‐weighting changes were stronger. In the dense‐tracking GRE and EPTI experiments, it also effectively reduced motion‐ and $\delta B_{0}$‐related artifacts.

Computationally, Aligned‐SENSE and Augmented‐SENSE apply a distinct encoding operator for each state during every reconstruction iteration. Mobile‐GRAPPA instead performs brief MLP training and a one‐time k‐space cleaning step before downstream reconstruction with approximately the no‐motion baseline complexity. For extreme motion, separate models can be trained for a small number of clusters, so the cost scales with the cluster count rather than the number of tracked states. No more than two clusters were required in the present experiments.

Because the preprocessing is separated from the downstream solver, the cleaned k‐space can be used with conventional CG‐SENSE, EPTI subspace reconstruction, or formulations with learned priors. In the clustered setting, the same principle is retained using a small number of intermediate states rather than all densely tracked states.

An important scope distinction is that Mobile‐GRAPPA targets pose‐ and respiration‐dependent $\delta B_{0}$ changes rather than the full static $B_{0}$ inhomogeneity. These dynamic changes are generally, spatially smooth and low dimensional and are therefore amenable to correction using local k‐space kernels. By contrast, particularly at long TE, static $B_{0}$‐induced phase can become substantially more spatially complex and may not be represented stably by strictly local operators.

Several limitations should be emphasized. First, Mobile‐GRAPPA remains dependent on the accuracy of the supplied motion and $\delta B_{0}$ estimates, and does not detect or correct tracking errors. In Supporting Information Figure S6, perturbing the supplied estimates degraded reconstruction quality, whereas a control scan without intentional motion produced only small deviations from the uncorrected reconstruction. Mobile‐GRAPPA showed sensitivity comparable to Aligned‐SENSE when the two methods were supplied with the same motion estimates. Second, residual differences in the stationary‐reference comparisons in Supporting Information Figures S2 and S3 may also reflect forward‐model limitations, including head‐orientation‐dependent changes in $T_{2}^{*}$ and susceptibility‐related signal phase that are not explicitly modeled.

Third, the calibration data are assumed to be motion‐free and artifact‐free. Motion or other inconsistencies during calibration could corrupt kernel training and are not addressed by the current framework. In addition, coil profiles are defined in scanner coordinates, which accounts for motion of the object relative to the assumed stationary receive coils, but pose‐dependent changes in the profiles caused by coil‐loading variation are not explicitly modeled. Finally, the need for clustering must currently be determined on a case‐by‐case basis because it depends on the motion range and the coil‐sensitivity variation. The $10^{\circ }$ rotational and 5‐mm translational ranges were established only for the 3T MPRAGE configuration in Experiment 1 using the 20‐channel head‐neck coil and should not be transferred directly to other configurations. At 7T, clustering may be required at smaller motion ranges, although the limited motion range in the present 7T dataset was adequately represented by a single MLP.

Future work should validate Mobile‐GRAPPA in larger and more diverse populations, including older adults and clinical populations with reduced ability to remain still. Another direction is to extend the method beyond rigid‐body motion by modeling spatially varying motion as locally rigid and augmenting the Mobile‐GRAPPA feature representation to describe nonrigid deformation.

## 6 Conclusions

Mobile‐GRAPPA provides a motion‐ and $\delta B_{0}$‐aware k‐space preprocessing framework that corrects trajectory perturbations, coil reweighting, and $\delta B_{0}$‐induced phase before downstream reconstruction. Its MLP‐parameterized local kernels incorporate dense tracking information through a one‐time cleaning step while preserving standard SENSE, subspace, and other reconstruction pipelines. A clustered variant extends the approach to extreme motion. Across 3T and 7T discrete‐state experiments, Mobile‐GRAPPA achieved image quality comparable to Aligned‐SENSE. In dense‐tracking experiments, it maintained reconstruction times close to the corresponding no‐motion baselines.

## Funding

This work was partially supported by R01MH116173, R01EB019437, R01HD114719, R21EB038677, and Siemens Healthineers.

## Conflicts of Interest

Daniel Polak and Stephen Cauley are employees of Siemens Medical Solutions USA. The remaining authors declare no conflicts of interest.

## Supporting information




**Figure S1:** Sensitivity of a single mobile‐GRAPPA model to rigid‐body motion range. Reconstructions and 5× absolute error maps relative to the corresponding simulated reference‐state reconstruction are shown for isolated rotations of 5° and 10° about the yaw, roll, and pitch axes and for isolated translations of 5 mm along the x, y, and z directions. Approx‐Aug‐SENSE and Mobile‐GRAPPA were evaluated under the same motion conditions, and all Mobile‐GRAPPA results were generated using a single MLP without clustering. Error maps are displayed using the same intensity scale. Red arrows indicate representative residual errors. **Figure S2:** Stationary‐reference validation using the 3D multi‐echo GRE protocol from Experiment 4 at 3T. (A) Motion and $\delta B_{0}$ estimates obtained every 8 TRs (360 ms) using SMENA, together with representative $\delta B_{0}$ maps. (B) Reconstructions at TE = 27 ms using no correction, Aligned‐SENSE compressed to 12 representative states, and Mobile‐GRAPPA using either 12 or all 1620 motion and $\delta B_{0}$ states. Reconstruction times and wholebrain NRMSE values relative to the stationary reference are reported above the images. Enlarged regions and red arrows highlight representative residual differences. **Figure S3:** Stationary‐reference validation using the 3D EPTI protocol from Experiment 5 at 3T. (A) Motion and $\delta B_{0}$ estimates obtained every 4 TRs (240 ms) using SMENA, together with representative $\delta B_{0}$ maps. (B) Reconstructions at TE = 25 ms using no correction, Aligned‐SENSE compressed to 12 representative states, and Mobile‐GRAPPA using either 12 or all 544 motion and $\delta B_{0}$ states. Reconstruction times and whole‐brain NRMSE values relative to the stationary reference are reported above the images. Enlarged regions and red arrows highlight representative residual differences **Figure S4:** Additional in vivo repetitions of Experiment 4 using the 3D multi‐echo GRE acquisition at 3T. (A) Motion trajectories and representative $\delta B_{0}$ maps are shown for three additional healthy volunteers with small, moderate, and large motion, respectively. (B) Reconstructions at TE = 27 ms using no correction, Aligned‐SENSE compressed to 12 representative states, and Mobile‐GRAPPA using all 1620 motion and $\delta B_{0}$ states. For the large‐motion case, full‐state Aligned‐SENSE using all 1620 states is additionally shown. Reconstruction times are reported above the images, and red arrows indicate representative residual artifacts or differences. **Figure S5:** Representative coil‐sensitivity magnitude maps and motion‐dependent $\delta B_{0}$ maps at 3T and 7T. (A) ESPIRiT‐derived magnitude sensitivity profiles from three representative receive coils at 3T and 7T, displayed on a normalized scale from 0 to 1. (B) Representative $\delta B_{0}$ maps from two motion states at 3T and 7T, displayed using the same frequency scale from −20 to 20 Hz. **Figure S6:** Sensitivity of Mobile‐GRAPPA to motion‐estimation accuracy. (A) Artificial perturbation of the motion estimates from Experiment 4. (i) The original estimates and one representative perturbed realization are shown. Independent zero‐mean Gaussian noise with a standard deviation of 0.2° for each rotational parameter and 0.2 mm for each translational parameter was added to generate five realizations. (ii) Reconstructions compare Aligned‐SENSE using the original estimates, compressed‐state Aligned‐SENSE and Mobile‐GRAPPA using the noisy estimates compressed to 12 representative states, and full‐state Aligned‐SENSE and Mobile‐GRAPPA using all 1620 noisy estimates. NRMSE values are reported as the mean and standard deviation across the five noise realizations. (B) Control scan without intentional motion. (i) Motion and $\delta B_{0}$ estimates obtained every 8 TRs (360 ms). (ii) Reconstructions using no correction, full‐state Aligned‐SENSE, and full‐state Mobile‐GRAPPA. NRMSE values for the corrected reconstructions are reported relative to the uncorrected reconstruction.

## Data Availability

The reconstruction code and example data for the Mobile‐GRAPPA pipeline are publicly available at https://github.com/linym20/Mobile-GRAPPA.
