# Supplementary material for: Fast Reconstruction of Motion‐Corrupted Data With Mobile‐GRAPPA: Motion and δB0 Inhomogeneity Correction Leveraging Efficient GRAPPA
Source: Magn Reson Med. 2026 Sep 4;97(1):70–89. doi: 10.1002/mrm.70575 (PMC13746106; doi:10.1002/mrm.70575)
Supplement: Supplementary file 1 [file MRM-97-70-s001.pdf]

A. Sensitivity of a single Mobile-GRAPPA model to rigid-body motion range

To examine the motion range over which one Mobile-GRAPPA model remains sufficient, isolated rigid-body transformations were retrospectively simulated about each rotational and translational axis. Rotations of 5° and 10° were applied about the yaw, roll, and pitch axes, and translations of 5 mm were applied along the x, y, and z directions. Approx-Aug-SENSE and Mobile-GRAPPA were evaluated under the same motion conditions. All Mobile-GRAPPA results in this experiment were generated using a single MLP without clustering. The corresponding simulated reference-state reconstruction before application of the rigid-body transformation served as the reference for the error maps.

As shown in Supporting Information Figure S1, residual errors were relatively modest for the 5° rotations. The errors became more apparent for the 10° rotations and for the 5-mm translations. The effect of translation was direction dependent, consistent with the spatially varying change in coil weighting produced by motion relative to the coils. These results characterize the empirical motion range represented by a single Mobile-GRAPPA model and motivate separate-model clustering when the within-cluster motion range approaches or exceeds approximately 10° in rotation or 5 mm in translation. These values should be interpreted as empirical guidelines for the tested 3T MPRAGE and 20-channel head-neck coil configuration rather than universal thresholds.

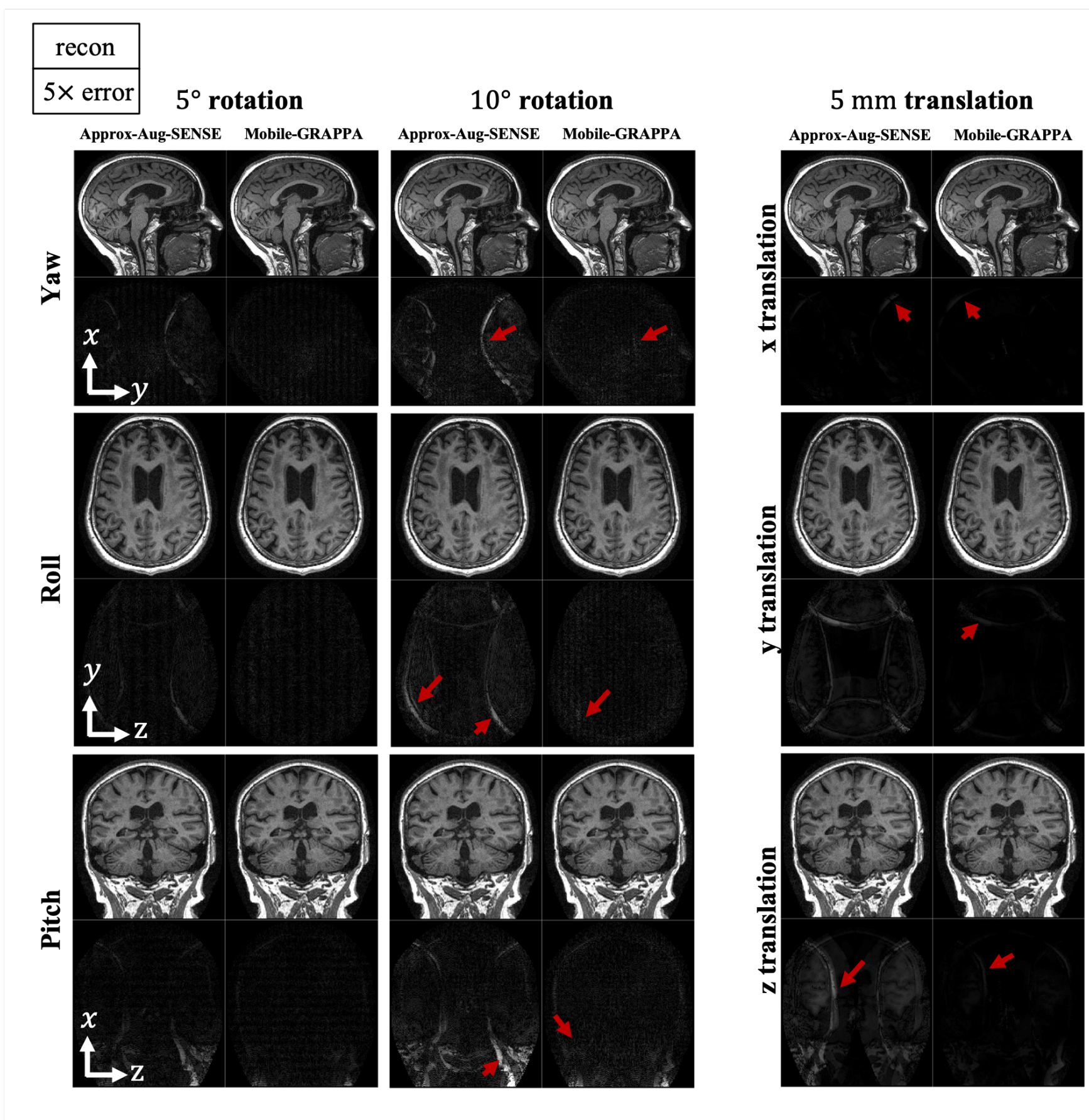

Figure S1: Sensitivity of a single Mobile-GRAPPA model to rigid-body motion range. Reconstructions and 5× absolute error maps relative to the corresponding simulated reference-state reconstruction are shown for isolated rotations of 5° and 10° about the yaw, roll, and pitch axes and for isolated translations of 5 mm along the x, y, and z directions. Approx-Aug-SENSE and Mobile-GRAPPA were evaluated under the same motion conditions, and all Mobile-GRAPPA results were generated using a single MLP without clustering. Error maps are displayed using the same intensity scale. Red arrows indicate representative residual errors.

B. Comparison with a stationary reference using Experiment 4 protocol

A stationary reference acquisition was used to assess the residual differences in the 3D multi-echo GRE experiment at 3T. The acquisition had 1-mm isotropic resolution and a scan time of 9.7 min. During the motion scan, SMENA provided motion and δB_0 estimates every 8 TRs, corresponding to a temporal resolution of 360 ms and a total of 1,620 states. Before NRMSE calculation, the stationary-reference reconstruction was rigidly registered to the reference pose of the motion scan. NRMSE was computed between magnitude images within a common object mask defined using an ESPIRiT eigenvalue threshold of 0.95.

Supporting Information Figure S2 compares no correction, Aligned-SENSE compressed to 12 representative states, Mobile-GRAPPA applied to 12 representative states, and Mobile-GRAPPA applied to all 1,620 states. Relative to the stationary reference, the whole-brain NRMSE values were 15.5% for no correction, 14.2% for state-compressed Aligned-SENSE, 14.4% for state-compressed Mobile-GRAPPA, and 14.3% for full-state Mobile-GRAPPA. Both state-compressed and full-state Mobile-GRAPPA achieved NRMSE values comparable to the state-compressed Aligned-SENSE benchmark. The similar results obtained with 12 representative states and all 1,620 states suggest that, for the relatively limited motion variation in this dataset, the compressed representation captured most of the motion and δB_0 variation relevant to reconstruction. However, all corrected reconstructions retained an approximately 14% difference from the separately acquired stationary reference. Because the stationary and motion scans were acquired separately, these residual differences may also include noise, physiological variation, and inter-scan differences and should not be interpreted as error relative to an absolute ground truth.

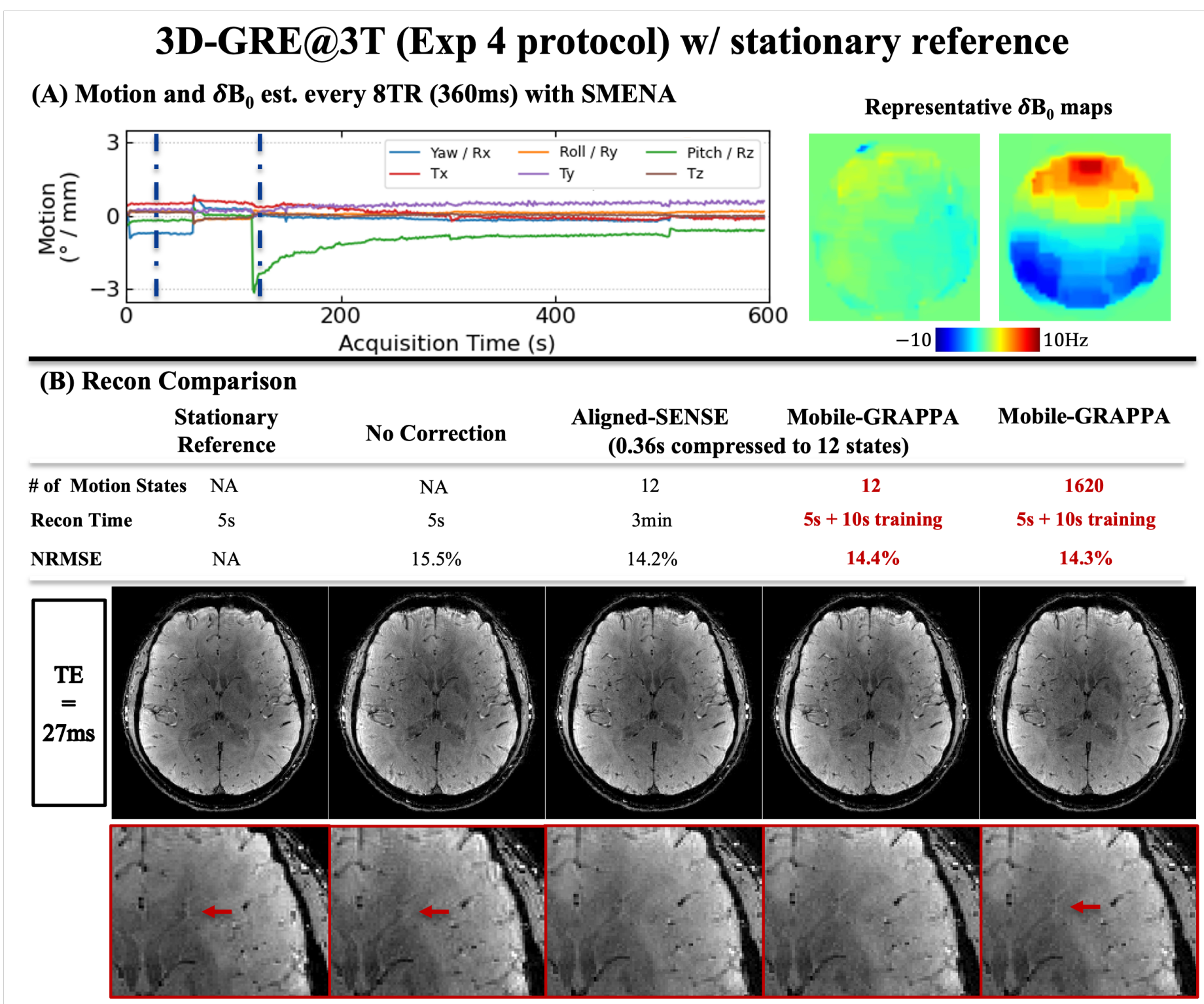

Figure S2: Stationary-reference validation using the 3D multi-echo GRE protocol from Experiment 4 at 3T. (A) Motion and δB_0 estimates obtained every 8 TRs (360 ms) using SMENA, together with representative δB_0 maps. **(B)** Reconstructions at TE = 27 ms using no correction, Aligned-SENSE compressed to 12 representative states, and Mobile-GRAPPA using either 12 or all 1,620 motion and δB_0 states. Reconstruction times and whole-brain NRMSE values relative to the stationary reference are reported above the images. Enlarged regions and red arrows highlight representative residual differences.

C. Comparison with a stationary reference using Experiment 5 protocol

The stationary-reference analysis was also performed for the 3D-EPTI acquisition at 3T. This acquisition had 1-mm isotropic resolution and a scan time of 2.2 min. Motion and δB_0 were estimated every 4 TRs, corresponding to a temporal resolution of 240 ms and a total of 544 states. The stationary-reference reconstruction was registered and the NRMSE was calculated using the same magnitude-image comparison and ESPIRiT-based masking procedure as in Supporting Information Figure S2.

As shown in Supporting Information Figure S3, the whole-brain NRMSE values relative to the stationary reference were 17.2% for no correction, 12.0% for Aligned-SENSE compressed to 12 representative states, 12.4% for 12-state Mobile-GRAPPA, and 11.7% for Mobile-GRAPPA using all 544 states. Thus, both correction approaches substantially reduced the difference from the stationary reference, and full-state Mobile-GRAPPA provided the lowest NRMSE among the motion-scan reconstructions while avoiding the long reconstruction time of Aligned-SENSE.

As in Supporting Information Figure S2, the separately acquired stationary scan serves as an independent reference rather than an absolute ground truth.

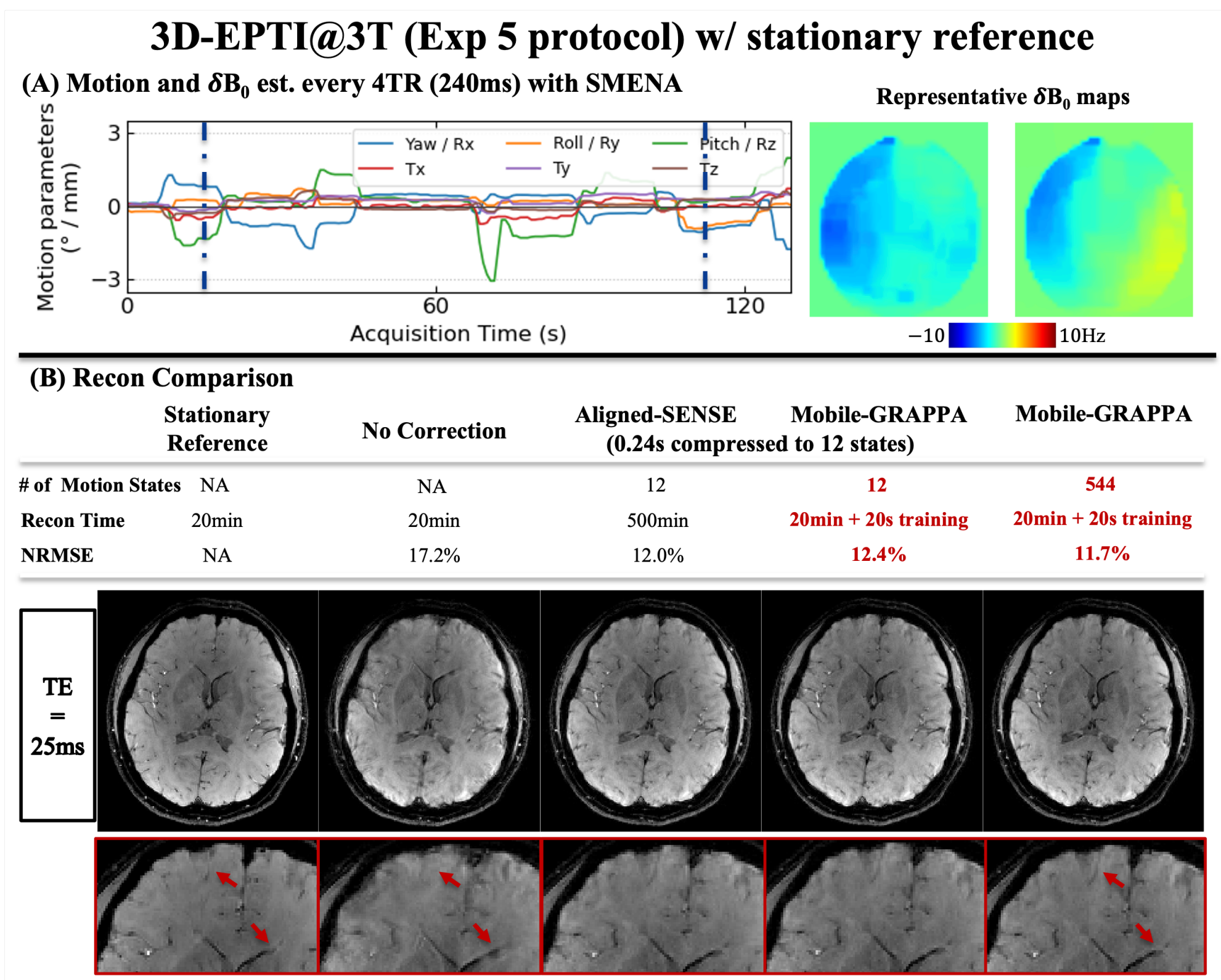

Figure S3: Stationary-reference validation using the 3D EPTI protocol from Experiment 5 at 3T. (A) Motion and δB_0 estimates obtained every 4 TRs (240 ms) using SMENA, together with representative δB_0 maps. **(B)** Reconstructions at TE = 25 ms using no correction, Aligned-SENSE compressed to 12 representative states, and Mobile-GRAPPA using either 12 or all 544 motion and δB_0 states. Reconstruction times and whole-brain NRMSE values relative to the stationary reference are reported above the images. Enlarged regions and red arrows highlight representative residual differences.

D. Additional repetitions of Experiment 4

To examine performance across subjects and motion patterns, Experiment 4 was repeated in three additional healthy volunteers. The acquisitions used the same 3D multi-echo GRE protocol at 3T with 1-mm isotropic resolution and a scan time of 9.7 min. The three datasets contained small, moderate, and large motion, respectively, with corresponding pose-dependent δB_0 changes.

Supporting Information Figure S4 shows that Mobile-GRAPPA reduced motion- and field-related artifacts across all three motion conditions. State-compressed Aligned-SENSE also improved image quality but required approximately 3 min per reconstruction, whereas Mobile-GRAPPA used all 1,620 states with approximately 10 s of one-time training and 5 s of reconstruction. In the large-motion dataset, Mobile-GRAPPA reduced the residual artifacts relative to state-compressed Aligned-SENSE, although some artifacts remained. Full-state Aligned-SENSE using all 1,620 states, which required approximately 600 min, exhibited similar residual artifacts. This comparison indicates that the residual errors were not specific to Mobile-GRAPPA, although it does not uniquely determine whether they arose from tracking errors, unmodeled motion-related effects, or other data inconsistencies.

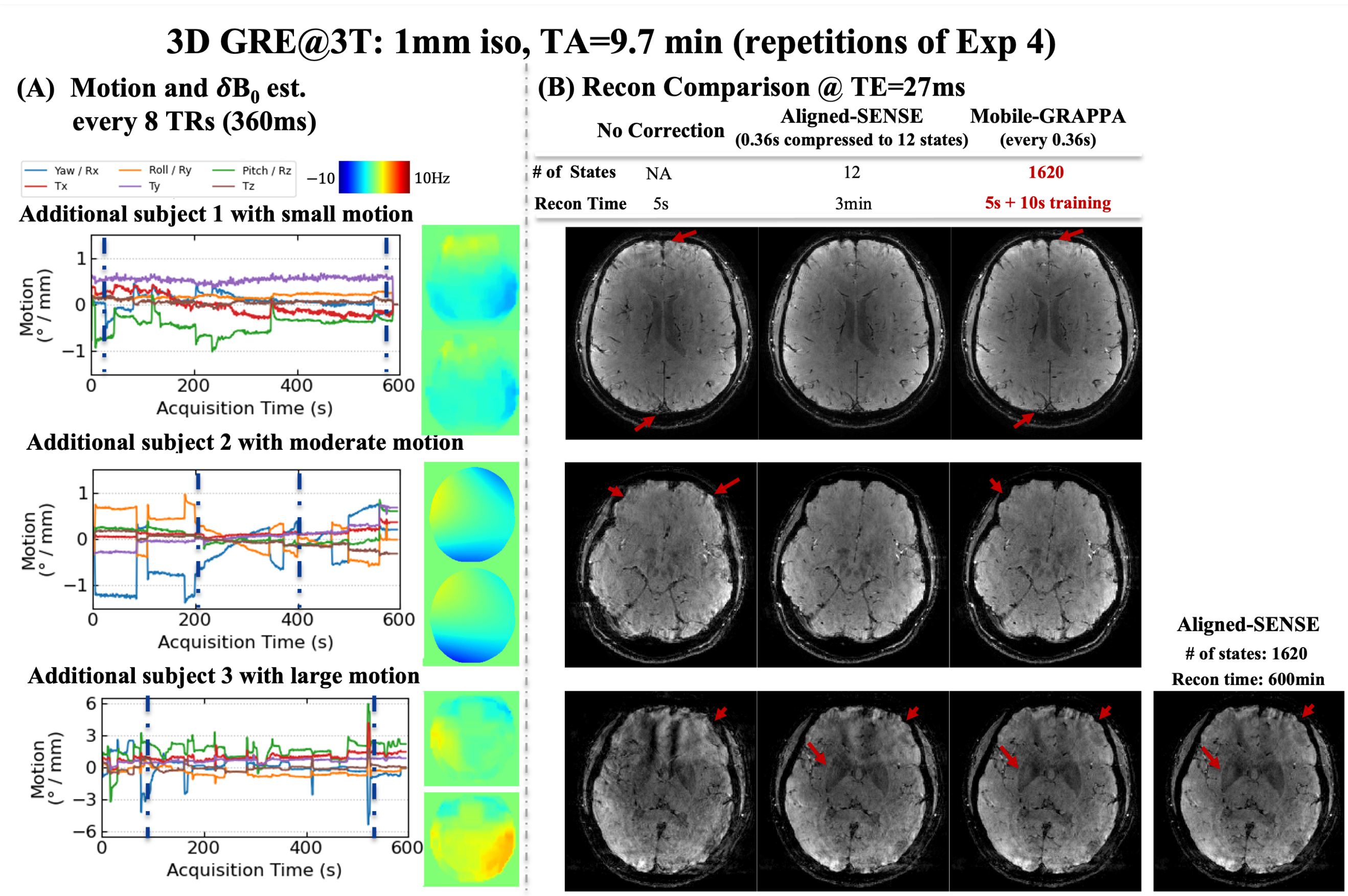

Figure S4: Additional in vivo repetitions of Experiment 4 using the 3D multi-echo GRE acquisition at 3T. (A) Motion trajectories and representative δB_0 maps are shown for three additional healthy volunteers with small, moderate, and large motion, respectively. **(B)** Reconstructions at TE = 27 ms using no correction, Aligned-SENSE compressed to 12 representative states, and Mobile-GRAPPA using all 1,620 motion and δB_0 states. For the large-motion case, full-state Aligned-SENSE using all 1,620 states is additionally shown. Reconstruction times are reported above the images, and red arrows indicate representative residual artifacts or differences.

E. Representative coil-sensitivity and δB_0 variation at 3T and 7T

Supporting Information Figure S5 qualitatively illustrates the coil-sensitivity and motion-dependent δB_0 patterns observed in the specific 3T (Experiment 2) and 7T (Experiment 3) experiments evaluated in this work. The representative 7T coil sensitivity magnitude maps appear more spatially localized than the corresponding 3T examples. Such spatially structured sensitivity profiles can make the effective coil weighting more sensitive to displacement of the head relative to the fixed coil array.

The representative motion-dependent δB_0 maps also show greater spatial variation at 7T than at 3T. Because the phase error produced by a frequency offset increases with echo time, these larger field perturbations can lead to stronger phase inconsistencies at 7T, particularly at longer echo times. The examples are intended to illustrate qualitative differences rather than provide a quantitative cross-field comparison.

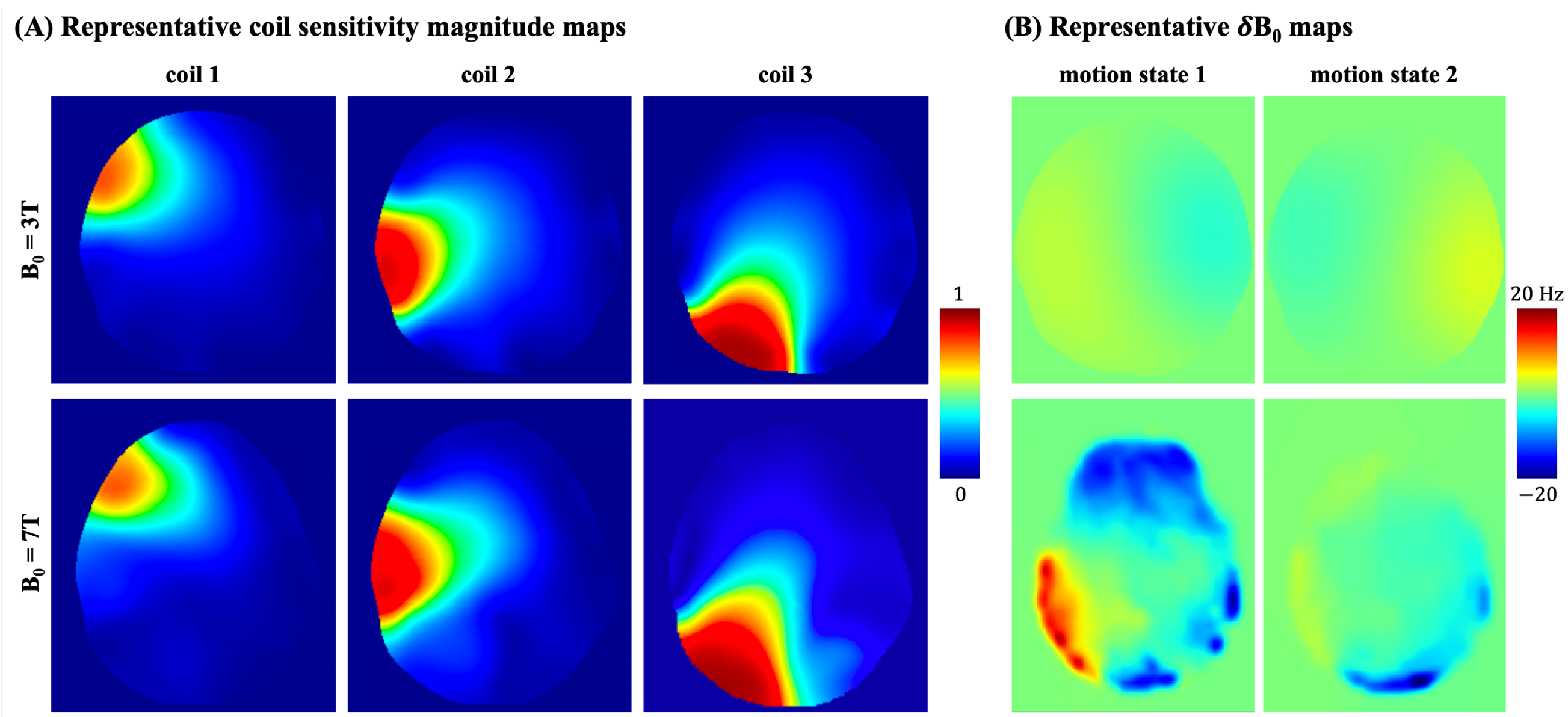

Figure S5: Representative coil-sensitivity magnitude maps and motion-dependent δB_0 maps at 3T and 7T. **(A)** ESPIRiT-derived magnitude sensitivity profiles from three representative receive coils at 3T and 7T, displayed on a normalized scale from 0 to 1. **(B)** Representative δB_0 maps from two motion states at 3T and 7T, displayed using the same frequency scale from −20 to 20 Hz.

F. Sensitivity to motion-estimation accuracy

The dependence of Mobile-GRAPPA on the supplied motion estimates was assessed using two complementary evaluations. First, the original motion estimates from Experiment 4 were independently perturbed with zero-mean Gaussian noise. The standard deviation was 0.2° for each rotational parameter and 0.2 mm for each translational parameter, and five independent noise realizations were generated. The corresponding δB_0 estimates were left unchanged. For each realization, we compared Aligned-SENSE using all 1,620 original estimates; state-compressed Aligned-SENSE and Mobile-GRAPPA using the perturbed estimates compressed into 12 representative states; and full-state Aligned-SENSE and Mobile-GRAPPA using all 1,620 perturbed estimates. The Aligned-SENSE reconstruction obtained using the original estimates served as a common comparison reference for the NRMSE calculation and was not assumed to be an artifact-free ground truth.

As shown in Supporting Information Figure S6A, perturbing the supplied motion estimates degraded the reconstruction quality for both methods. Across the five noise realizations, Aligned-SENSE and Mobile-GRAPPA using 12 representative noisy states yielded NRMSE values of $14.2\% \pm 0.38\%$ and $14.5\% \pm 0.37\%$, respectively. When all 1,620 perturbed estimates were retained, the corresponding NRMSE values were $20.1\% \pm 0.10\%$ and $19.7\% \pm 0.06\%$. Thus, Mobile-GRAPPA and Aligned-SENSE exhibited comparable sensitivity when supplied with the same state representation. Under the tested perturbation level, the full-state reconstructions showed higher NRMSE than the corresponding state-compressed reconstructions, consistent with more direct propagation of sample-wise errors in the supplied motion estimates. This observation should not be interpreted as evidence that state compression generally corrects tracking errors; rather, the experiment characterizes sensitivity to motion-estimation error.

Second, a control acquisition was performed without intentional motion. The estimated motion remained small, with rotational fluctuations of approximately $\pm 0.3^\circ$ and translational fluctuations of approximately ± 0.3 mm. Supporting Information Figure S6B compares no correction, full-state Aligned-SENSE, and full-state Mobile-GRAPPA using all 1,620 estimated states. Relative to the uncorrected reconstruction, Aligned-SENSE and Mobile-GRAPPA produced NRMSE values of 3.5% and 3.9%, respectively. The uncorrected reconstruction was used only as a comparison reference and was not assumed to be an artifact-free ground truth. The similar deviations produced by the two methods indicate that Mobile-GRAPPA responds to the supplied residual motion and δB_0 estimates comparably to Aligned-SENSE, while neither method can determine whether those estimates are themselves accurate.

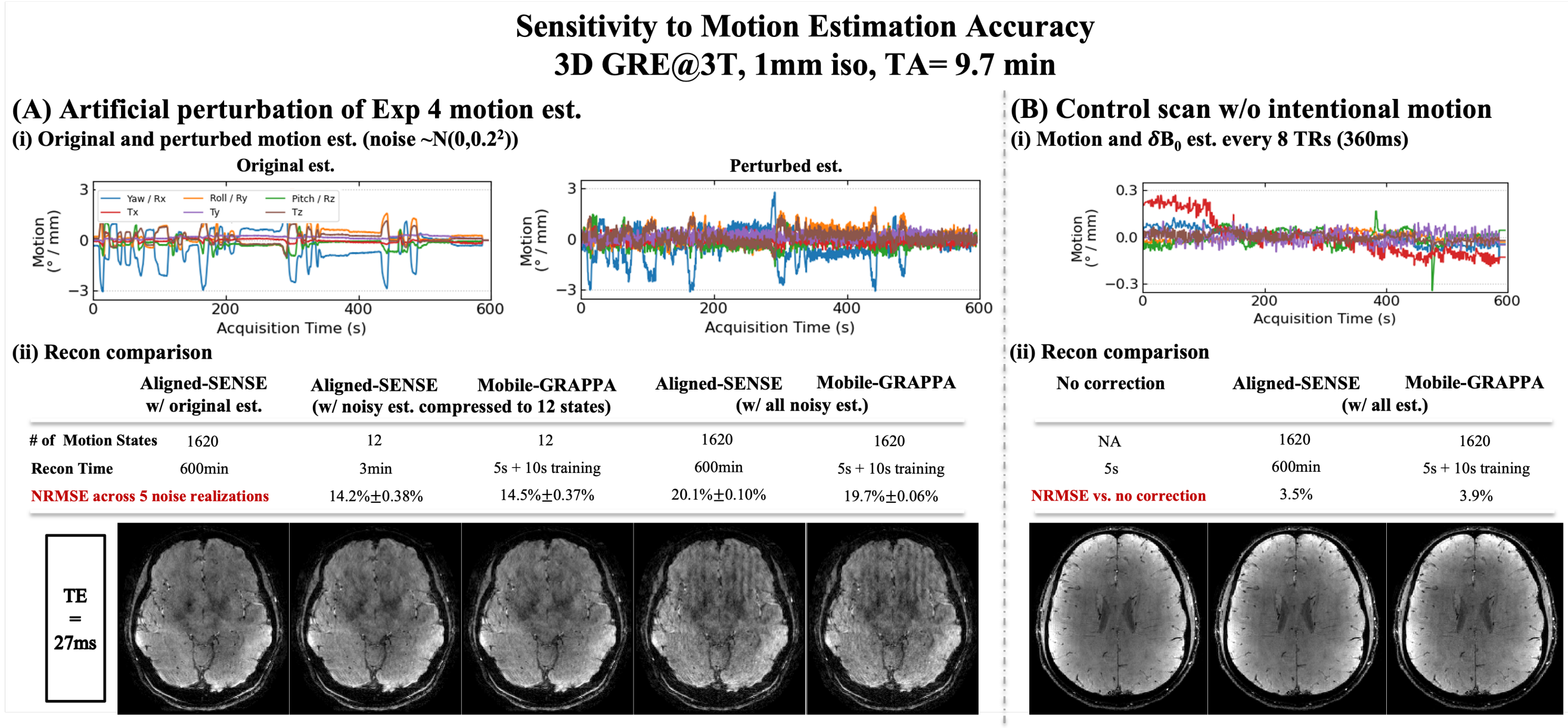

Figure S6: Sensitivity of Mobile-GRAPPA to motion-estimation accuracy. **(A)** Artificial perturbation of the motion estimates from Experiment 4. **(i)** The original estimates and one representative perturbed realization are shown. Independent zero-mean Gaussian noise with a standard deviation of 0.2° for each rotational parameter and 0.2 mm for each translational parameter was added to generate five realizations. **(ii)** Reconstructions compare Aligned-SENSE using the original estimates, compressed-state Aligned-SENSE and Mobile-GRAPPA using the noisy estimates compressed to 12 representative states, and full-state Aligned-SENSE and Mobile-GRAPPA using all 1,620 noisy estimates. NRMSE values are reported as the mean and standard deviation across the five noise realizations. **(B)** Control scan without intentional motion. **(i)** Motion and δB_0 estimates obtained every 8 TRs (360 ms). **(ii)** Reconstructions using no correction, full-state Aligned-SENSE, and full-state Mobile-GRAPPA. NRMSE values for the corrected reconstructions are reported relative to the uncorrected reconstruction.
